# Full‐Body AI Agent: A Perspective on Multi‐Scale Collaborative AI for Systemic Biology and Precision Medicine

**DOI:** 10.1002/advs.202520562

**Published:** 2026-05-26

**Authors:** Aoqi Wang, Jiajia Liu, Jianguo Wen, Yangyang Luo, Zhiwei Fan, Liren Yang, Xi Hu, Ruihan Luo, Yankai Yu, Sophia Li, Weiling Zhao, Xiaobo Zhou

**Affiliations:** ^1^ West China Biomedical Big Data Centre West China Hospital Sichuan University Chengdu Sichuan P. R. China; ^2^ Center For Computational Systems Medicine McWilliams School of Biomedical Informatics The University of Texas Health Science Center at Houston Houston Texas USA

**Keywords:** AI agent, multi‐agents system, systems biology

## Abstract

Artificial intelligence (AI) is increasingly applied to biomedical research, but most current systems remain limited to specific tasks, data types, or biological scales. This makes it difficult to connect molecular alterations, organelle dysfunction, cellular behavior, tissue remodeling, organ physiology, systemic regulation, and whole‐body phenotypes into coherent biological reasoning. In this Perspective, we propose the Full‐Body AI Agent as a hypothetical multi‐agent framework and conceptual blueprint for future systemic biology and precision medicine, rather than a fully implemented software platform. This framework envisions a supervisory Full‐Body AI Agent coordinating seven biological‐level agents, namely Molecule, Organelle, Cell, Tissue, Organ, Organ System, and Body System AI Agents, to standardize biomedical data, decompose cross‐scale questions, assign level‐specific tasks, and integrate outputs through iterative feedback. We further outline the data commons, harmonization mechanisms, uncertainty handling, arbitration strategies, and traceability safeguards required for biologically grounded cross‐scale reasoning. Two hypothetical scenarios, metastasis analysis and drug development, illustrate how this framework could organize multilevel evidence from molecular changes to systemic phenotypes and therapeutic responses. This Perspective aims to clarify the conceptual basis of full‐body AI and provide a foundation for transparent, physiology‐constrained, cross‐scale AI systems in disease analysis, therapeutic evaluation, and personalized medicine.

## Graphical abstract

We propose the Full‐Body AI Agent, a multi‐scale collaborative framework with 7 biological‐layer agents. It unifies multi‐omics/clinical data via standardized protocols, enabling phenotype‐guided closed‐loop reasoning, quantitative evaluation, and LLM safeguards, with promising applications in tumor metastasis modeling and precision drug development.

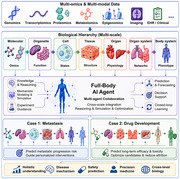

## 1 Introduction

Human biology is characterized by intricate, multilevel interactions that span from molecular processes to the functioning of entire organ systems [1]. Historically, biological research primarily focused on isolated biological processes or systems. Questions were centered on understanding the function of individual genes or proteins, or investigating the role of specific cells in disease. While these approaches were essential for advancing basic biological knowledge, their limitations became evident as they were unable to fully capture the complexity of interactions among diverse biological effects [2].

With the emergence of Artificial Intelligence (AI) and its integration into systems biology, the landscape of biological research has changed significantly [3]. AI's ability to process large datasets, uncover hidden patterns, and predict outcomes has helped to overcome many limitations of traditional research methods [4]. For instance, deep learning algorithms have enabled researchers to analyze complex biological data at unprecedented scales and depths. These advancements have had a profound impact on various fields. In cancer diagnostics and treatment [5, 6, 7], AI has improved diagnosis accuracy and the effectiveness of treatment planning. In clinical genetics [8, 9, 10], it has facilitated the identification of disease‐associated genetic mutations. In drug discovery and development [11, 12], AI has accelerated the identification of new drug candidates and the optimization of existing ones. Furthermore, transformative progress in epidemiology and public health [13, 14], as well as evolutionary biology and phylogenetic studies [15, 16], has been driven by synergistic advances in both experimental innovations and computational algorithm development. One example is the Evo2 framework, a novel deep‐learning architecture that integrates phylogenetic inference with population genetic simulations to resolve ancestral selection patterns, achieving an 87% higher resolution in detecting ancestral selection patterns compared to conventional methods [17].

Today, biological research increasingly focuses on multi‐scale questions, such as how genetic mutations at the molecular level influence cellular processes, how cellular behaviors shape tissue development, and how disturbances at tissue or organ levels propagate into systemic diseases [18, 19]. Although AI‐driven approaches have enhanced our ability to analyze multi‐omics data and biological interactions [20], methodological constraints remain. These limitations hinder the completeness and interpretability of multi‐scale information extraction [21]. Despite advances in computing and health information technology providing new sources of biological data, the collection of comprehensive multi‐scale patient data in real‐world clinical settings remains costly and challenging [22]. Therefore, a more integrated approach is needed. This approach should combine data‐driven methods, biologically informed modeling, and advanced tools such as Large Language Models (LLMs) to better understand and predict the behavior of complex biological systems across multiple scales. LLM can decompose high‐level tasks into sub‐goals, reason through problems step‐by‐step in a manner similar to the human chain of thought, and plan sequential actions to achieve defined objectives [23]. While LLMs hold significant promise for integrating multi‐scale biological data due to their capability in capturing complex interactions, key challenges remain, particularly regarding biological interpretability and the effective harmonization of heterogeneous data sources [24].

AI Agents, autonomous computational systems, offer an innovative solution for tackling complex biological tasks [25]. They can adaptively perform multiple tasks, interpret diverse data modalities, make informed decisions, and interact with dynamic environments. By integrating into research and clinical workflows, AI Agents enable efficient, scalable, and precise analysis across various biological domains, enhancing both scientific discovery and clinical decision‐making. Some AI agents have already been successfully deployed. For example, ChatGPT's Advanced Data Analysis (ADA) can independently process real‐world clinical datasets from different medical specialties [26]. ADA is capable of autonomously developing and implementing machine learning models, streamlining complex analytical workflows, and reducing the need for extensive human intervention. Another notable example is nuclei.io, a pathologist‐AI system that significantly improves diagnostic accuracy in pathology slide analysis while enabling the creation and utilization of autonomous tools [27]. Additionally, SpatialAgent integrates dynamic tool execution and self‐directed reasoning to autonomously perform end‐to‐end tasks in spatial biology research from experimental design to multimodal data analysis, including the identification of cell interaction networks, spatial metabolic microenvironments, and disease biomarkers [28]. Such advances highlight the potential of AI Agents to enhance both efficiency and accuracy in biological research and clinical practice. Moreover, multiple AI Agents can collaborate synergistically to enable the integration of multi‐omics and multimodal data, facilitating comprehensive biological insights and precision medicine applications, as exemplified by the Virtual Cell Framework initiative [29]. This integrated approach allows for a unified analysis of heterogeneous datasets, cross‐modal validation, and system‐level modeling of biological processes, ultimately bridging the gap between molecular mechanisms and clinical outcomes. For example, Robin is a multi‐agent AI system that fully automates literature search, hypothesis generation, experimental design, and data analysis in a closed‐loop workflow, culminating in the discovery and validation of ripasudil as a novel therapy for dry age‐related macular degeneration [30].

Here, we propose a Full‐Body AI Agent Framework designed to tackle the multi‐scale challenges in biological research. The core concept is that complex biological problems can be decomposed into manageable tasks, each handled by specialized AI agents working collaboratively. In this Perspective, the Full‐Body AI Agent is not presented as a fully implemented software platform, but as a conceptual and operational blueprint for future multi‐agent systems in systemic biology and precision medicine. This framework is conceptualized as a supervisory Full‐Body AI Agent coordinating multiple collaborative basic AI Agents, including the Molecule AI Agent, Organelle AI Agent, Cell AI Agent, Tissue AI Agent, Organ AI Agent, Organ System AI Agent, and Body System AI Agent, to model biological processes across different hierarchical scales. By simulating interactions among these agents, the Full‐Body AI Agent facilitates a comprehensive understanding of how changes at one biological level propagate to others, providing valuable insights into disease mechanisms, treatment responses, and personalized medicine. At the heart of the framework is a modular, collaborative network of AI Agents, with each basic AI Agent dedicated to a specific biological level. These agents communicate via standardized data exchange protocols, facilitating dynamic and systemic modeling of human biology. By bridging molecular mechanisms with higher‐order physiological processes, the framework is designed to support comprehensive research in disease analysis (such as tumor metastasis), drug development, and precision medicine. We use tumor metastasis and drug development as illustrative scenarios to show how the Full‐Body AI Agent concept could support hypothesis generation and cross‐scale task organization.

**FIGURE 1:**
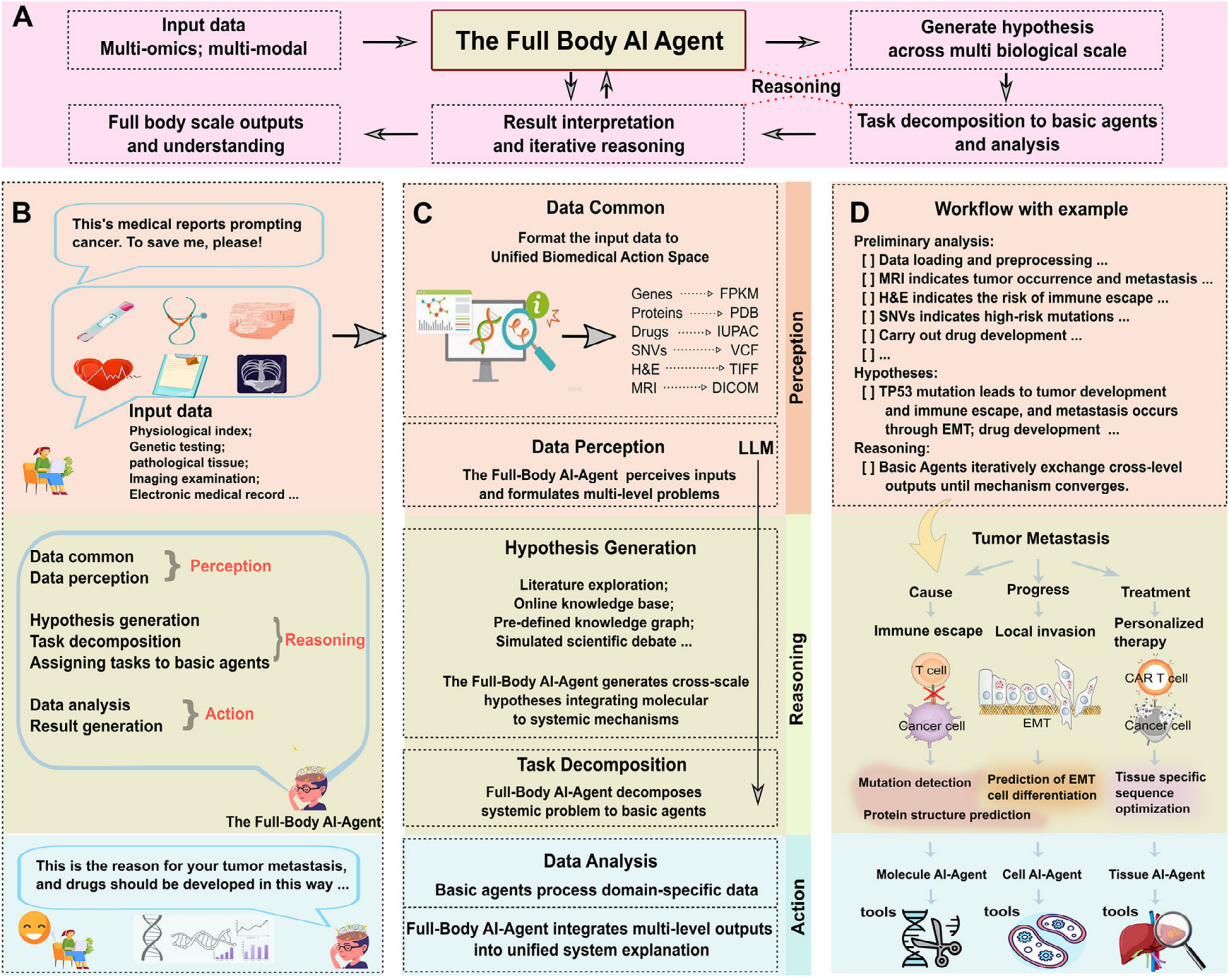
Schematic of the Full‐Body AI Agent framework for systemic human biology analysis and therapeutic insight generation. (A) presents the core reasoning cycle of the Full‐Body AI Agent, where multi‐modal and multi‐omics data are input to the central supervisor agent, which perceives and preprocesses data, generates cross‐scale biological hypotheses, decomposes tasks to level‐specific basic agents for analysis, and then integrates and iteratively refines results to deliver a unified, systemic interpretation. (B) illustrates the patient‐centric data input and interaction workflow, where diverse clinical inputs, including physiological indices, genetic testing, pathological tissue, imaging examinations, and electronic medical records, are ingested, enabling the agent to translate raw data into actionable, patient‐specific insights and therapeutic recommendations. (C) details the LLM‐driven reasoning pipeline, structured into three interconnected stages: a Data Common layer standardizes heterogeneous data into a unified biomedical action space for multilevel problem perception; the agent generates cross‐scale hypotheses by leveraging literature, knowledge bases, and simulated scientific debate, then decomposes systemic tasks to basic agents; and these basic agents execute domain‐specific data analysis, with the Full‐Body AI Agent integrating multilevel outputs into a cohesive system explanation. (D) demonstrates the framework in an oncology use case centered on TP53 mutation‐driven tumor metastasis, where preliminary analysis of multi‐modal data (MRI, H&E, SNVs) identifies high‐risk mutations and metastatic potential, hypotheses are generated linking TP53 mutation to tumor development, immune escape, and metastasis, the agent decomposes tasks to basic agents (molecule, cell, tissue) for mutation detection, EMT prediction, and tissue‐specific sequence optimization, and integrated results enable mechanistic modeling to guide personalized therapeutic strategies such as CAR‐T cell therapy.

As illustrated in Figure 1A, the Full‐Body AI Agent framework is proposed as an integrated conceptual architecture for organizing multi‐modal and multi‐omics biological data across scales. The Full‐Body AI‐Agent, functioning as a supervisor agent, perceives and preprocesses data to generate cross‐scale biological hypotheses. It decomposes cross‐scale biological problems, assigning tasks and data specific to biological levels to basic agents. These basic agents act as executors, perceiving data at specific biological levels, further refining tasks, and conducting data analysis. The Full‐Body AI‐Agent integrates the results from each basic agent and can iteratively use conclusions from one biological level as input for other levels, repeating the reasoning process to capture cross‐biological insights. The framework begins with a patient interacting with the Full‐Body AI Agent (as shown in Figure 1B), which processes diverse inputs, including physiological indices, genetic tests, pathological images, and clinical records, through a structured workflow: perceiving data, reasoning, and action to analyze by basic agents. The system handles multiple data modalities, including text (i.e., genomic sequences, Electronic Medical Record (EMRs)), images (i.e., MRI (Magnetic Resonance Imaging), CT (Computed Tomography), H&E (Hematoxylin and Eosin)), and numerical data (i.e., RNA‐seq, physiological signals), mapping them across biological levels from molecules to the whole body. This enables the modeling of the entire disease continuum, from causation (i.e., mutation detection, protein structure prediction) and progression (i.e., immune escape, epithelial–mesenchymal transition (EMT), invasion) to treatment (i.e., personalized therapy, CAR‐T (Chimeric Antigen Receptor T‑cell) cell optimization). Specialized basic agents operate at each biological level, while a collaborative network works through standardized data exchange protocols. This design enables the dynamic, systemic modeling of human biology, revealing how changes at one level propagate across scales, thereby informing disease understanding and therapeutic development. Figure 1C demonstrates the significance of LLM in the Full‐Body AI Agent system, which runs through the entire reasoning process, including data perception, hypothesis generation, and task decomposition, and uses the literature Knowledge bases and scientific debate models are established to ensure the generation of valuable cross‐level biological insights. Furthermore, Figure 1D illustrates a conceptual workflow using the hypothesis that TP53 mutation may contribute to tumor development, immune evasion, and metastasis via EMT as an example. The Full‐Body AI Agent perceives the data, decomposes it into biological tasks across different levels, and prompts basic agents to perform mutation detection, protein structure prediction, and prediction of EMT cell differentiation. Additionally, it guides tissue‐specific sequence optimization in drug design. We compared the Full‐Body AI Agent system with recent multi‐agent systems (i.e., OriGene [31], Robin [30], Biomni [32], MAC‐doctor [33], PharmaSwarm [34], and AI Co‐Scientist [35]) and provided a detailed description of the analytical approach for systemic human biology problems (i.e., metastasis research and drug development), thereby informing disease understanding and therapeutic development.

## 2 Inter‐Level Data Commons and Integration of Biomedical Data

**FIGURE 2:**
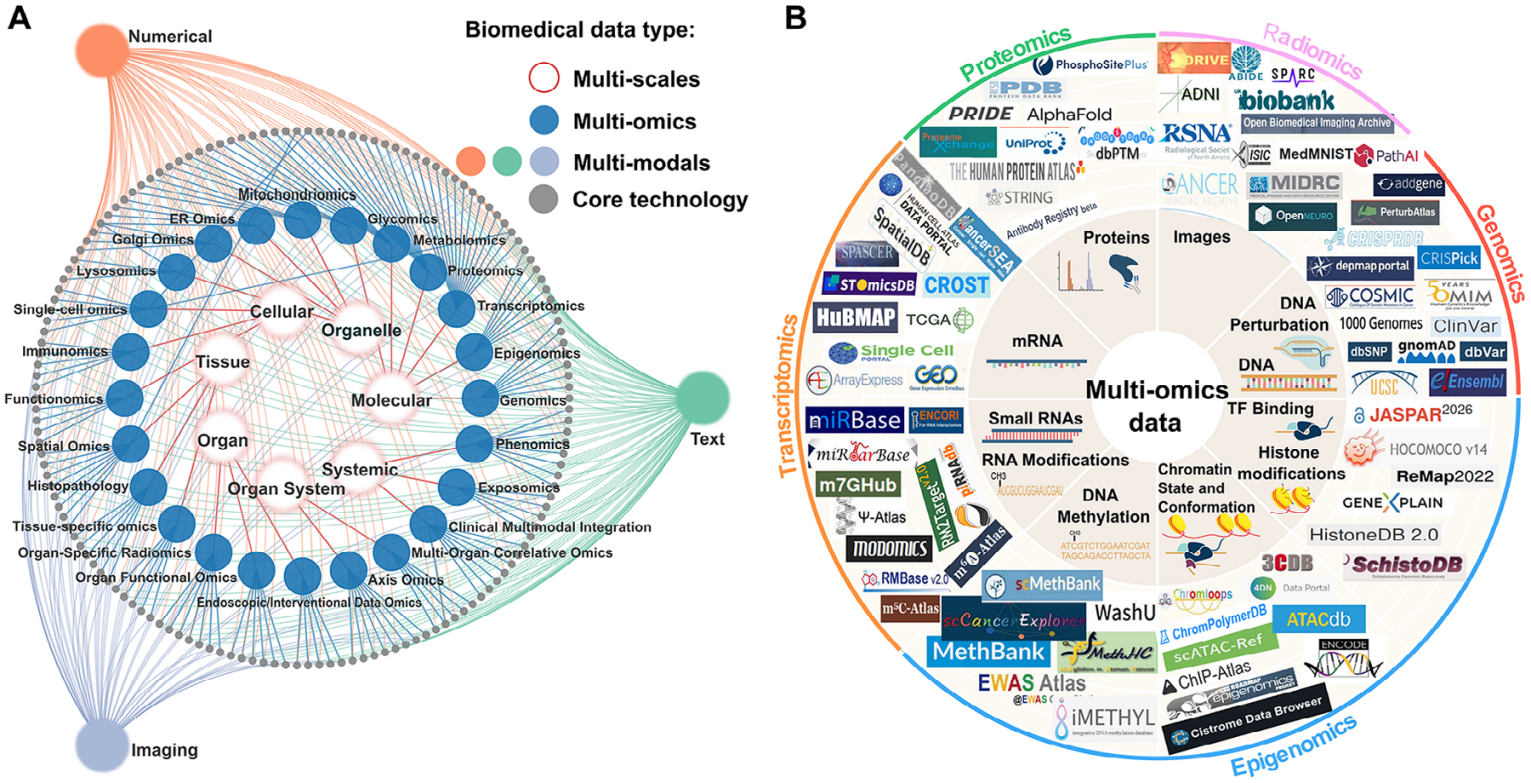
Overview of multi‐modal and multi‐omics of biomedical data types. (A) Schematic representation of data modalities and their integration across seven hierarchical biological scales (Cellular, Organelle, Molecular, Tissue, Organ, Systemic, and Organ System). The network illustrates 24 distinct omics categories (blue nodes) and their connections to three primary data modalities: Numerical (orange), Imaging (blue), and Text (green), with approximately 150 non‐redundant biotechnologies represented. Red lines highlight multi‐scale connections, while gray nodes denote core enabling technologies. (B) Overview of publicly accessible biomedical databases supporting multi‐omics data integration. The circular diagram categorizes 105 databases by major data domains, including Genomics, Epigenomics, Transcriptomics, Proteomics, and Radiomics, with key examples (e.g., TCGA, PRIDE, RSNA, HuBMAP). The central “Multi‐omics data” hub emphasizes the integration of diverse data types (e.g., DNA, mRNA, proteins, images) and modalities (bulk, single‐cell, spatial) to enable multi‐scale biological reasoning.

The landscape of biomedical research is defined by an explosion of multi‐modal, multi‐omics data that spans biological scales, from the molecular precision of genomics and proteomics to the macroscopic insights of medical imaging and clinical phenotypes [36, 37]. While this data ecosystem is transformative, it remains inherently fragmented. Each dataset is characterized by distinct formats, acquisition protocols, and analytical pipelines. Figure 2A illustrates the diversity and complexity of multimodal and multi‐omics biomedical data, and Figure 2B lists 105 databases corresponding to different omics. One effective approach to addressing this challenge is to establish data standards and perform standardized pre‐analysis to meet the scalability requirements of modern research. A prominent example of a data commons is Bioteque, which provides standardized low‐dimensional embeddings of over 450,000 biomedical entities and 30 million relationships from more than 150 sources, enabling efficient machine learning applications [38]. (The data in Figure 2A are stored in Table S1, which records multiple omics and multimodal data for each biological level, along with the corresponding experimental techniques. The data in Figure 2B is stored in TableS2, which includes 105 databases and access links by major data domains, such as Genomics, Epigenomics, Transcriptomics, Proteomics, Radiomics, and the corresponding databases for clinical phenotypic data.)

In the context of the Full‐Body AI Agent framework, establishing a robust interlevel data ecosystem is crucial for the future operationalization of cross‐scale biological reasoning. This can be achieved through the implementation of a Data Commons, adherence to Common Format standards, and integration with advanced language models. The Data Commons serves as a shared repository, acting as the central hub for curating a diverse range of biomedical datasets. It operates under a unified set of rules and guidelines, enabling the seamless aggregation of data from various sources across different biological levels.

Before defining the Data Commons, it is important to clarify why the Full‐Body AI Agent organizes biomedical information into seven biological levels. This organization is not proposed as an empirically optimized architecture, nor do we claim that it is superior to simpler orchestration schemes. Rather, it reflects how biomedical data, tools, and research questions are currently fragmented in practice. Genomic variants, protein structures, and pathway annotations are typically analyzed at the molecular level; mitochondrial activity, nuclear damage, and other subcellular processes are studied at the organelle level; scRNA‐seq and snRNA‐seq data describe cell states and cell‐type composition; spatial transcriptomics and histopathology characterize tissue architecture; radiology and physiological measurements capture organ‐level structure and function; immune, endocrine, circulatory, respiratory, and metabolic indicators describe organ‐system regulation; and electronic health records, longitudinal imaging, treatment response, recurrence, survival, and patient‐reported outcomes define body‐level phenotypes. Although each data type is powerful within its own scale, these resources are usually stored in different formats, processed by different tools, and interpreted within different biological assumptions. As a result, a molecular finding may remain disconnected from cell‐state change, tissue organization, organ physiology, systemic regulation, and clinically actionable phenotype. The seven‐layer structure is therefore used as a practical organizing schema for the inter‐level Data Commons. In this sense, the Data Commons is not merely a storage repository, but a cross‐scale translation layer that helps convert isolated biological observations into phenotype‐oriented reasoning chains. The practical value of this seven‐level organization relative to simpler orchestrators, fewer‐module systems, or direct multimodal models remains to be tested in future benchmarked implementations.

### 2.1 Data Commons and Common Format

The Data Commons is established with standard protocols to manage data across molecular, cellular, tissue, organ, and system levels. Its effectiveness is closely tied to the implementation of Common Format standards. Together, Data Commons and Common Format standards allow data from diverse sources and biological levels to be accurately interpreted, efficiently processed, and seamlessly integrated by various components of the Full‐Body AI Agent framework. This Data Common lays a solid foundation for hierarchical data mapping and multi‐scale data interactions. Table S3, titled “Multi‐omics and Multi‐modal Data Mapping to Biological Hierarchies” includes columns such as Biological Hierarchy, Omics/Modalities/Levels, Data Types, Common Data Storage Formats, Data Preprocessing Methods, and Data Commons. It serves as a cornerstone of the Full‐Body AI Agent framework, providing critical infrastructure to bridge disparate biological data types across scales and enable holistic, multilevel modeling of human biology. This table indicates that through standard protocols, heterogeneous data across genomics, epigenomics, proteomics, imaging, and clinical records can be systematically organized into a structured hierarchy (from molecules to organ systems) to facilitate the integration of data with different morphologies, resolutions, and biological backgrounds. By defining standardized data types, storage formats (e.g., FHIR (Fast Healthcare Interoperability Resources), DICOM (Digital Imaging and Communications in Medicine), H5AD (Hierarchical Data Format 5 with AnnData)), preprocessing methods (e.g., FastQC for quality control, Seurat for single‐cell clustering), and Data Commons repositories for each biological level, the table ensures that data from siloed domains (e.g., molecular sequencing, organ imaging, patient EMRs) can be harmonized, interoperable, and computationally tractable for the AI Agent ecosystem.

### 2.2 Data Integration Across Biological Levels

At the molecular level, the Data Commons stores genomic sequences, proteomic profiles, and biochemical pathways. Resources such as GenBank and the Protein Data Bank (PDB) contribute standardized datasets, ensuring consistency in nucleotide sequences, protein structures, and functional annotations across studies. These data adhere to widely used formats, FASTA for nucleotide sequences and PDB for protein structures, which facilitate seamless data exchange and analysis. Within this framework, the Data Commons resources and Common Format standards for each biological level are documented to support consistent data access and integration. LLMs such as the Evo and Evo 2 genome language models use standardized resources in the Data Commons to align sequences, variants, and functional annotations to the Common Format and to produce unified representations that enable consistent retrieval, cross‐study comparison, and downstream analysis [17, 39].

At the cellular level, the Data Commons integrates information from single‐cell sequencing and imaging, including common cell type annotations, unified expression profiles, and standardized cellular markers. To enable effective aggregation and comparison, this data is formatted according to established standards that bioinformatics tools can readily process. BioLLM provides a unifying framework for integrating and benchmarking heterogeneous single‐cell foundation models, enabling harmonized cell type annotations, unified expression profiles, and standardized marker sets within a common schema that downstream tools can process efficiently [40]. scGPT supplies pretrained representations learned from tens of millions of cells and supports label transfer, batch robust integration, perturbation reasoning, and multi‐omics alignment, yielding consistent embeddings for retrieval and cross‐study comparison [41]. And, to connect computational curation with experimentation, an LLM and object detection‐enhanced active matrix digital microfluidics platform enables programmatic single‐cell manipulation and links provenance and feedback to the commons [42].

At the tissue and organ levels, the Data Commons houses histopathological and radiological data. Universal imaging protocols, spatial mapping coordinates, and tissue‐specific biomarkers ensure data consistency, while the DICOM standard for medical images ensures compatibility across different imaging modalities and devices, enabling seamless data integration and interpretation. Within this standardized layer, SpaCCC and QuST‐LLM support spatial transcriptomics by inferring cell–cell communication, aligning spot‐level expression with tissue architecture, and exporting harmonized coordinates for downstream analysis [43, 44]. A vision language foundation model for precision oncology links histopathology images with textual knowledge to support biomarker discovery and report generation [45]. In radiology, LLM‐based workflows map narrative reports to structured DICOM‐compatible representations and normalize imaging ontologies, enabling queryable views that interoperate with molecular and cellular evidence [46].

At the system level, clinical data is incorporated into the Data Commons. Standardized using clinical terminologies and Electronic Medical Record (EMR) protocols, this data is formatted to align with the overall framework, supporting interoperability and comprehensive analysis. Clinical data originate from hospital information systems, including Clinical Information Systems, Laboratory Information Systems, Picture Archiving and Communication Systems, Radiology Information Systems, and Operating Room Information Systems, and public resources such as MIMIC (Medical Information Mart for Intensive Care), the eICU (electronic Intensive Care Unit) Collaborative Research Database, and HiRID (High‑frequency Intensive Care Data) provide curated cohorts. Interoperability and semantic consistency are ensured by controlled vocabularies such as ICD 10 (International Classification of Diseases), SNOMED CT (Systematized Nomenclature of Medicine Clinical Terms), and LOINC (Logical Observation Identifiers Names and Codes), with records structured according to the HL7 FHIR (Health Level Seven; Fast Healthcare Interoperability Resources) specification and commonly stored as JSON, XML, or Turtle; a typical JSON record contains a resource type, an identifier, and key patient fields. Medical large language models trained on clinical language processed EMR content to analyze the chief complaint and context, recommend evidence‐based examinations, propose differential diagnoses, and articulate stepwise clinical reasoning, with MedQA (Medical Question Answering) serving as a widely used benchmark for training and evaluation [47].

By integrating into the Full‐Body AI Agent, these standardized data streams form queryable and semantically aligned longitudinal records that support hypothesis generation, validation, and cross‐scale integration with omics and imaging, improving diagnostic accuracy and enabling a systemic view of human biology.

### 2.3 Cross‐Scale Data Conflict Reconciliation

Cross‐layer communication in biological reasoning systems cannot rely on direct information transfer, since data generated at molecular, cellular, tissue, and organ levels differ substantially in measurement principles, sampling density, and uncertainty structure [48]. Single‐cell transcriptomic measurements are inherently sparse, with observed zeros reflecting a mixture of biological absence and technical dropout effects, leading to zero‐inflated count structures that distort downstream inference if treated naively [49]. Spatial omics technologies often operate at spot‐level or probe‐limited resolution, such that measurements represent mixtures of multiple cell states rather than true single‐cell profiles, constraining the granularity of downstream reasoning [50]. And, at the organ level, imaging modalities capture macroscopic physiological states that are shaped by modality‐specific acquisition biases and reconstruction artifacts, introducing uncertainty structures fundamentally different from molecular measurements. Suggest that explicit coupling operators that transform representations, reconcile scale mismatches, and propagate uncertainty rather than merely transferring raw observations across layers.

To enable coherent cross‐scale reasoning, the Full‐Body AI‐Agent framework suggests introducing harmonization operators that translate outputs between biological levels through biologically meaningful intermediate representations rather than propagating raw data. For example, single‐cell outputs can be denoised and aggregated into mesoscale descriptors such as pathway activity, lineage composition, or cell‐state abundance, allowing tissue‐level agents to reason about spatial dynamics without relying on high‐dimensional matrices. Conversely, tissue‐level imaging or spatial omics signals are transformed into region‐specific functional descriptors (e.g., perfusion capacity or mechanical stiffness) that support organ‐level reasoning while preserving physiological interpretability. Recent multimodal foundation‐model studies suggest that learned shared representations may provide a practical route for harmonizing heterogeneous biological modalities across scales [50]. In this context, harmonization functions as a mechanism for constructing biologically grounded equivalence mappings across scales, allowing heterogeneous observations to participate in unified reasoning processes.

Because biological observations are acquired at incompatible spatial and temporal resolutions, cross‐scale reasoning requires explicit resolution‐matching modules that align sampling granularities before integration [50]. Fine‐grained molecular or cellular outputs are aggregated into regional summaries using spatial or anatomical priors that match tissue‐level granularity, while coarse organ‐level observations are mapped onto anatomically constrained regions of interest to guide downstream reasoning. Such resolution alignment can be operationalized through region‐based aggregation, spatial registration, or graph‐structured anatomical mapping, enabling information transfer across scales while preserving biological topology [48]. In parallel, uncertainty propagation ensures that each agent's output carries confidence descriptors reflecting measurement noise and sampling limitations, enabling confidence‐aware integration rather than assuming homogeneous data reliability.

Cross‐scale communication is inherently bidirectional: higher‐level agents generate constraint signals that are translated into biologically meaningful priors guiding lower‐level inference, enabling top–down refinement rather than unidirectional aggregation. Conflicts between layers are resolved through arbitration mechanisms that compare competing hypotheses according to their uncertainty estimates and consistency with higher‐level physiological constraints. Rather than forcing immediate consensus, the supervisory agent maintains alternative explanations and selectively requests refinement from agents whose predictions show lower confidence or conflict with cross‐scale evidence. In this way, arbitration acts as a confidence‐guided reconciliation process that stabilizes iterative feedback while preserving biologically plausible uncertainty. Through harmonization, resolution matching, uncertainty propagation, bidirectional feedback, and arbitration‐driven integration, cross‐layer communication becomes a controlled reasoning process that maintains physiological consistency while enabling scalable multi‐scale biological inference. Together, these mechanisms frame cross‐layer communication as a controlled and context‐aware reasoning process that supports physiologically consistent multi‐scale inference.

Recent biomedical AI systems have begun to move toward cross‐scale collaborative architectures. For example, emerging spatial‐biology agent frameworks combine image analysis, spatial omics, and downstream biological inference through modular agent coordination, representing early implementations of cross‐scale AI reasoning in biomedical research [51].

## 3 Reasoning of the Full‐Body AI‐Agent Framework

**FIGURE 3:**
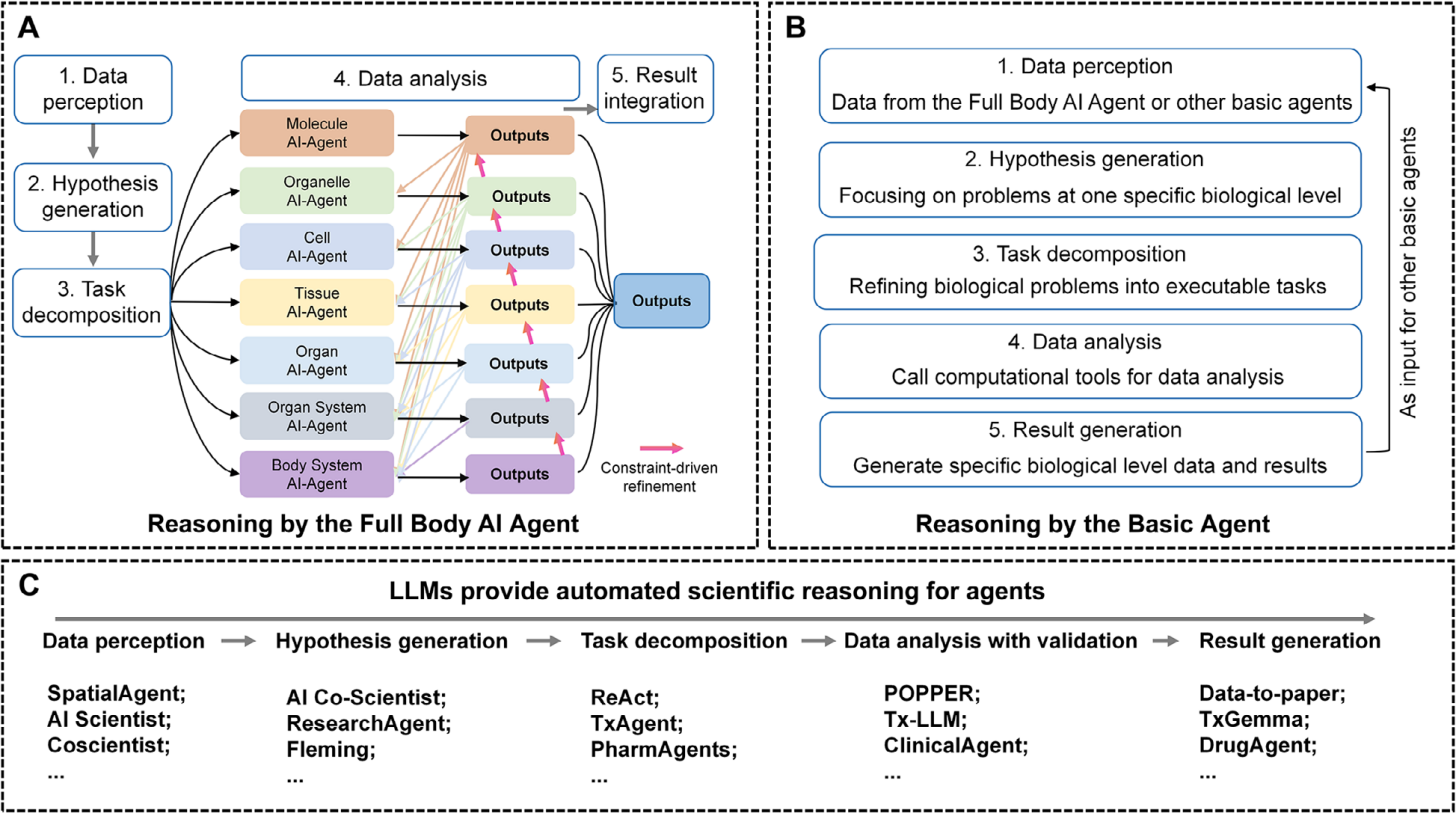
Hierarchical reasoning framework of Full‐Body and basic AI Agents for multi‐scale biological discovery. (A) The Full‐Body AI Agent orchestrates a closed‐loop scientific reasoning workflow, initiating with data perception and hypothesis generation, then decomposing tasks across seven hierarchical biological AI Agents (molecule, organelle, cell, tissue, organ, organ system, and body system) for parallel data analysis. The framework incorporates a constraint‐driven refinement mechanism, where high‐level agent outputs (e.g., body system‐level conclusions) feedback as prior knowledge to refine low‐level agent operations, culminating in integrated, multi‐scale results. (B) Each Basic AI Agent operates within a dedicated biological level, executing a focused reasoning cycle: it accepts input data (from the Full‐Body Agent or other basic agents), generates targeted hypotheses, decomposes tasks into executable steps, calls specialized computational tools for data analysis, and outputs level‐specific results that can be used as input for other agents. (C) A modular LLM toolchain underpins the entire reasoning pipeline, with specialized agents (e.g., SpatialAgent, AI Co‐Scientist, ReAct, TxAgent, ClinicalAgent, TxGemma) supporting distinct stages from data perception and hypothesis generation to task decomposition, validated data analysis, and final result generation, enabling end‐to‐end systems biology discovery.

The reasoning mechanism of the Full‐body AI‐Agent operates through a structured pipeline: raw multi‐modal data is first standardized for compatibility; complex biological questions and data are decomposed into hierarchical sub‐tasks aligned with specific biological levels; these sub‐tasks are assigned to corresponding specialized AI Agents, which execute them using domain‐specific tools; and results are integrated via iterative feedback loops, enabling bidirectional cross‐scale information flow to reveal multilevel biological associations. Here, Figure 3A illustrates the overview of the core components of the reasoning system of the Full‐Body AI‐Agent architecture. Upon receiving biological data, the Full‐Body AI‐Agent perceives data (1), generates a hypothesis (2), performs task decomposition (3), assigns subtasks to basic agents, and uses domain‐specific tools to analyze data (4), optimizes and synthesizes outputs (5). Similar to the Full‐body AI‐Agent, basic agents have a similar reasoning structure. The difference is that basic agents perceive and execute inputs and tasks at a specific biological level (as shown in Figure 3B).

The proposed reasoning process of the reasoning process of the Full‐body AI‐Agent and basic agents could be supported by multiple LLMs (as shown in Figure 3C). In this Perspective, we describe how hierarchical reasoning in LLMs and existing agent frameworks could be organized to support a future closed‐loop scientific workflow spanning data perception, hypothesis generation, task decomposition, data analysis, validation, and result generation.

Operationally, the Full‐Body AI Agent routes data perception to Coscientist for technical documentation and assay protocol parsing [23], to AI Scientist for literature ingestion and dataset onboarding [52], to SpatialAgent for multimodal tissue images and spatial omics [28], and to TxAgent adapters for structured clinical and biomolecular endpoints [53]. Hypotheses are proposed and ranked by AI Co‐Scientist [35] and ResearchAgent [54]. Task decomposition follows a ReAct planning policy [55], and TxAgent composes the required tools and protocols for each subtask, PharmAgents scales the plan across preclinical and clinical handoffs [56], and DrugAgent generates executable simulation and ADMET (Absorption, Distribution, Metabolism, Excretion, and Toxicity) pipelines that can be audited and reused [57]. In drug development tasks, the Full Body AI Agent also consults Fleming [58] to propose candidate chemotypes and optimization directions. Data analysis and validation are carried out by Tx‐LLM [59] and TxGemma [60] for therapeutic property prediction and design time analytics, by ClinicalAgent [61] for trial information extraction and inference, and under a POPPER [62] sequential falsification loop that enforces pre‐specified tests and error control. When performing large‐scale data analysis, the Full Body AI Agent can trigger automated experiments through Coscientist or use Fleming to connect design decisions to screening outcomes. Result generation is handled by Data‐to‐Paper [63], which renders traceable methods, results, and figures from the executed analyses, and by Tx‐LLM when regulatory style summaries are needed for downstream decision making.

Conceptually, the Full Body AI Agent binds reasoning to explicit biological levels and enforces bidirectional constraints across scales. For example, claims at the organ level must be supported by tissue and cellular signals, while lower‐level findings are checked against organism‐level plausibility. Cross‐scale routing aligns hypotheses, plans, and analyses with the biological level where they are most informative. Provenance complete outputs make the workflow auditable and repeatable. In effect, the architecture turns a catalogue of capable agents into a coherent operating system for multilevel biology.

### 3.1 Data Input and Standardization for Perception

The Full‐body AI‐Agent Framework begins by receiving raw biological data along with a corresponding research problem or question provided by the user. These inputs may include genomic sequences, clinical observations, imaging results, and experimental datasets, often presented in diverse formats. The system first perceives and interprets these inputs, parsing the raw data and extracting the core biological question embedded in the user's inquiry. Once the problem is identified, the framework proceeds to standardize the data to ensure consistency and compatibility across the entire system. This involves cleaning raw data to remove inconsistencies, resolve missing values, and reduce noise, thereby enhancing data quality for further analysis. The standardized data is then converted into a structured, unified format, making it assignable and interpretable by specialized AI agents at various biological levels. This process ensures that data from various sources and formats can be used seamlessly in downstream tasks, transforming the user's input into a precise biological question that can be tackled effectively by the system.

### 3.2 Hypothesis Generation and Task Decomposition

Once the biological problem and corresponding data are standardized, the Full‐body AI Agent initiates the critical step of task decomposition. This process involves breaking down a complex biological question into smaller, more manageable sub‐tasks aligned with specific biological levels. The decomposition process ensures that each part of the problem is addressed with the appropriate focus and depth, allowing for specialized analysis at each biological level. The decomposition follows a hierarchical structure, ensuring that all layers of the biological system are considered and tasks are tackled using domain‐specific expertise. This enables the Full‐body AI Agent to tackle biological complexity using a layered and modular approach. Each sub‐task is clearly defined to address the unique challenges of its corresponding biological level, while maintaining coherence with the overarching research objective. This structured decomposition also allows for parallel execution of tasks at different levels, enabling more efficient problem‐solving while maintaining the integrity of the biological model. At this stage, the Full‐body AI‐Agent identifies the fundamental biological components involved in the problem and divides them into sub‐tasks based on biological layers. To facilitate effective task execution, we define structured biological components at each biological level based on data modes and research content, so that AI agents can fully understand requirements and perform tasks. We define a hierarchical design of collaborative AI‐Agents as a foundational reference for implementing the Full‐body AI‐agent system. The following are the task assignments for the main levels.

#### 3.2.1 Molecular‐Level Tasks

These tasks focus on the genetic, epigenetic, transcriptomic, proteomic, and biochemical foundations of biological processes. They include analyzing genetic variants, gene regulation, protein structure and function, protein–protein interactions, biochemical pathways, and molecular signaling. At this level, the goal is to identify key molecular perturbations, characterize their functional consequences, and define the upstream mechanisms that may influence higher biological levels.

#### 3.2.2 Organelle‐Level Tasks

These tasks focus on subcellular structures and organelle‐specific functions, such as the nucleus, mitochondria, endoplasmic reticulum, Golgi apparatus, lysosomes, and peroxisomes. They include analyzing mitochondrial metabolism, oxidative stress, apoptosis, DNA damage response, protein processing, organelle morphology, and inter‐organelle communication. At this level, the goal is to determine how molecular alterations affect organelle function and how organelle dysfunction contributes to cellular state changes.

#### 3.2.3 Cellular‐Level Tasks

At the cellular level, the framework decomposes tasks related to cell states, behaviors, and interactions. These tasks include studying gene expression regulation, cell signaling, proliferation, apoptosis, differentiation, migration, immune evasion, stress responses, and cell‐cell communication. Cellular analysis is essential for understanding how molecular and organelle‐level alterations manifest as functional changes in individual cells or cell populations.

#### 3.2.4 Tissue‐Level Tasks

Building on cellular‐level insights, tissue‐level tasks focus on how coordinated cellular behaviors give rise to tissue organization and pathological remodeling. These tasks include modeling tissue architecture, spatial cell distribution, extracellular matrix remodeling, immune infiltration, hypoxia, vascular niches, and tissue‐specific responses to injury or disease. Tissue‐level analysis helps explain how altered cell states become spatially organized into functional or pathological tissue contexts.

#### 3.2.5 Organ‐Level Tasks

Building on tissue‐level evidence, organ‐level tasks focus on the structure, function, and pathological changes of individual organs. These tasks include analyzing organ anatomy, organ‐specific physiology, lesion progression, blood flow, nutrient transport, local immune responses, functional impairment, and organ‐specific treatment responses. At this level, the goal is to determine how tissue‐level alterations affect organ function and disease progression within a specific anatomical context.

#### 3.2.6 Organ‐System‐Level Tasks

At the organ‐system level, tasks address the coordination and communication among multiple organs within a physiological system, such as the immune, circulatory, respiratory, nervous, endocrine, digestive, or metabolic system. These tasks include modeling inter‐organ signaling, systemic inflammation, immune surveillance, circulatory dissemination, endocrine regulation, metabolic coordination, and multi‐organ pathological cascades. This level is crucial for understanding how local organ changes propagate through connected physiological systems.

#### 3.2.7 Body‐System‐Level Tasks

At the whole‐body level, tasks focus on organism‐level phenotypes, clinical outcomes, and integrated physiological states. These tasks include analyzing disease progression, treatment response, recurrence, survival, comorbidities, patient‐reported outcomes, longitudinal clinical records, and whole‐body homeostasis. At this level, the goal is to integrate evidence from all lower biological levels into a coherent patient‐level interpretation for disease mechanism analysis, therapeutic evaluation, and precision medicine.

### 3.3 Task Assignment to AI Agents to Analyze Data

Decomposed tasks are assigned to the corresponding AI Agents specialized at each biological level. Each agent is responsible for domain‐specific analysis using its specialized knowledge and tools. This hierarchical approach is intended to support tasks being handled at the relevant biological level while maintaining cross‐level communication. Once assigned, each AI Agent further refines its tasks into actionable and measurable sub‐tasks. These sub‐tasks are designed to be linked to appropriate data types, computational tools, and validation criteria. Figures S1–S7 illustrate the functional areas that need to be addressed at each biological level, as well as the process of task refinement rather than evidence of implemented execution. These figures use a consistent visual template to facilitate comparison across the Molecule, Organelle, Cell, Tissue, Organ, Organ System, and Body System AI Agents, while each figure represents a distinct biological task space. Agents then employ relevant biological tools and computational models to support their assigned tasks. These tools include sequencing techniques, image analysis software, simulation models, or other domain‐specific technologies. For example, the Cell AI Agent may use single‐cell RNA tools, such as scDRS [64], to study disease‐associated profiles for cells, whereas the Organ AI Agent may use imaging data to assess organ structure and function.

### 3.4 Feedback and Result Integration

After each AI Agent generates level‐specific outputs, the resulting outputs are transmitted to other agents operating at different biological levels. These outputs serve as inputs for further analysis, allowing the next level of agents to generate new insights based on the initial findings. This inter‐agent communication facilitates a multilevel refinement of the biological model. Finally, all outputs are returned to the Full‐body AI‐Agent, where they are integrated through an iterative feedback loop. This process is intended to refine the aggregated results by accounting for dynamic interactions across molecular, cellular, tissue, organ, and systemic levels. The iterative nature of this loop supports continuous model refinement and the generation of more biologically consistent interpretations. Once the outputs are integrated and synthesized, the Full‐body AI Agent presents the synthesized results to the user. The user can then select specific outputs based on the biological context or research objectives. These selected outputs are then reintroduced into the system for further refinement and deeper analysis of the biological phenomena across all levels. This user‐guided selection provides focused exploration of specific aspects of the biological system, facilitating the derivation of comprehensive, interpretable insights.

### 3.5 Toward Quantitative Evaluation of Hierarchical Reasoning

Recent advances in medical AI agents demonstrate that structured agent‐based systems can be quantitatively evaluated through operational metrics rather than solely task‐specific accuracy [65]. For example, large medical language models such as Med‐PaLM2 have been assessed using standardized clinical benchmarks [66], while hospital‐deployed AI monitoring systems have evaluated stability and reliability through prospective performance measures during continuous patient monitoring [67]. More broadly, emerging clinical AI agents integrate literature knowledge, guidelines, and real‐world patient data to support multi‐step reasoning, where performance is increasingly assessed through consistency, robustness, and longitudinal reliability rather than isolated prediction accuracy. These developments suggest a broader shift toward evaluating agent systems at the level of reasoning processes, including task decomposition, evidence integration, and feedback stability.

Building on this direction, the Full‐Body AI‐Agent is conceptualized as a biology‐constrained conceptual–operational framework that organizes multi‐agent reasoning across molecular, cellular, tissue, organ, and system scales. Rather than representing a finalized implementation, the framework serves as an operational blueprint in which biological structure guides task decomposition, cross‐scale information exchange, and hierarchical inference. Scientific questions can be mapped onto biologically meaningful task progressions, while intermediate representations enable translation between heterogeneous data modalities and preserve physiological interpretability throughout the reasoning process. In this view, biological knowledge provides structural constraints that maintain coherence across scales while allowing flexible data‐driven exploration. For example, model‐interpretability studies such as ChatNT illustrate how attribution methods can be used to examine whether model predictions rely on biologically coherent features [68].

From an operational perspective, this framework also suggests a pathway toward measurable system‐level evaluation of hierarchical reasoning. Future implementations could assess task decomposition fidelity by examining whether reasoning trajectories follow biologically expected transitions, and evaluate cross‐scale alignment by testing consistency between intermediate representations across adjacent biological levels. In addition, conflict–resolution effectiveness may be quantified by examining whether arbitration among heterogeneous agent outputs improves coherence, while end‐to‐end predictive stability can be monitored through convergence behavior during iterative feedback cycles. Together, these evaluation dimensions provide a quantitative lens for assessing hierarchical multi‐agent reasoning. (The system‐level quantitative evaluation framework, detailed in Table S4, is proposed to assess the fidelity, stability, and convergence of hierarchical multi‐agent reasoning within the Full‐Body AI‐Agent architecture.)

### 3.6 LLM Safeguards and Traceability Mechanisms

Although large language models enable flexible coordination across biological agents, multi‐hop reasoning increases the risk that unsupported intermediate claims propagate across scales [69]. For example, Med‐PaLM 2 achieved strong but imperfect performance on medical question‐answering benchmarks, with a best‐reported accuracy of 86.5% on MedQA, indicating that even specialized medical LLMs retain non‐negligible error rates [47]. More importantly, in a realistic clinical decision‐making benchmark based on 2400 MIMIC patient cases, Hager et al. showed that LLMs performed significantly worse than physicians across four abdominal pathologies and degraded further when required to gather diagnostic information themselves. In that autonomous setting, diagnostic accuracy decreased from 58.8% to 45.5% for Llama 2 Chat, from 67.8% to 54.9% for OASST, and from 65.1% to 53.9% for WizardLM [70]. The same study also found that models made instruction‐following errors every two to four patients and hallucinated nonexistent tools every two to five patients. These findings are directly relevant to cross‐scale biological reasoning, because a mistaken tool call, unsupported molecular interpretation, or unverified intermediate claim could be propagated into misleading tissue‐, organ‐, or patient‐level conclusions. To reduce hallucination accumulation, the Full‐Body AI‐Agent framework introduces several operational safeguards.

First, LLM modules are restricted to orchestration tasks and must invoke predefined computational tools through schema‐constrained interfaces [71]. Each invocation requires structured inputs and returns validated outputs, preventing language models from generating unsupported quantitative or mechanistic conclusions directly. Second, cross‐scale reasoning is governed by explicit validation steps inserted between reasoning hops [72]. After each agent interaction, outputs undergo ontology‐consistency checks and measurement‐context validation to ensure compatibility with biological scale constraints. Predictions that violate schema definitions, conflict with physiological rules, or lack supporting tool‐derived evidence are flagged and excluded from downstream propagation. This step prevents local hallucinations from cascading into higher‐level reasoning. Third, all interactions are recorded within a traceable reasoning graph [73]. Each node stores the initiating agent, invoked tool, transformation operation, and associated uncertainty profile, allowing complete reconstruction of reasoning trajectories. When inconsistencies emerge, the supervisory agent can localize the origin of error and trigger targeted re‐execution rather than repeating the entire reasoning chain. Together, tool‐grounded execution, inter‐hop validation, and trace‐based rollback establish a practical safeguard mechanism that limits hallucination accumulation while preserving interpretability and reproducibility across multi‐scale biological reasoning.

To mitigate hallucination accumulation and ensure traceability in future multi‐hop, cross‐scale reasoning, we propose a standardized cross‐layer data‐flow protocol (Table S5; including Table S5a for Cross‐layer data objects, Table S5b for Harmonization operators, and TableS5c for Physiology‐first arbitration protocol), which operationalizes data harmonization, conflict resolution, and provenance tracking across all biological levels within the Full‐Body AI‐Agent framework.

### 3.7 Comparison of Multi‐AI Agent Systems in Biomedical Research

Current multi‐agent biomedical AI systems, such as OriGene [31], Robin [30], Biomni [32], MAC‐doctor [33], PharmaSwarm [34], and AI Co‐Scientist [35], are generally optimized for specific stages of the scientific workflow or confined to particular biomedical subdomains. Their reasoning processes tend to be scale‐bounded and follow largely linear trajectories: information is retrieved or computed, analyzed within a single domain, and then aggregated at a final integration stage. While these architectures achieve notable efficiency and accuracy within their niches, including molecular target discovery, literature synthesis, and clinical diagnostics, they rarely model causal propagation across biological levels. Table S6 makes a detailed comparison of the workflows of these multi‐agent systems.

Reasoning serves as the core module of an agent, responsible for transforming evidence into mechanisms and hypotheses. OriGene exemplifies template‐driven reasoning, in which a coordinator interprets a query, assigns tasks, and a reasoning component assembles mechanistic evidence into candidate targets, followed by post‐hoc validation. Robin implements a closed‐loop workflow that repeatedly queries literature and re‐computes analyses, but its feedback loops remain confined to the same scale of inquiry. Biomni's reasoning is predominantly procedural, centred on generating and executing analysis code across a large curated toolbox, with refinements occurring within a fixed data scale. MAC applies supervised consensus‐building among domain‐specialist “doctor” agents, integrating their interpretations at the clinical scale without modelling upstream molecular or tissue‐level causality. PharmaSwarm distributes hypothesis generation across agents focused on omics, literature, and market intelligence, reconciling outputs through cross‐source corroboration rather than multi‐scale constraint. AI Co‐Scientist employs iterative epistemic refinement through hypothesis generation, critique, ranking, and evolution, but its reasoning loop primarily optimises explanatory coherence rather than enforcing consistency across biological hierarchies.

The Full‐Body AI Agent is not intended to replace existing biomedical agents. Its added contribution is a biology‐constrained coordination layer for organizing existing and future tools around cross‐scale biological questions. Specifically, it contributes phenotype‐oriented task decomposition, cross‐scale routing of outputs between biological levels, physiology‐first constraint checking, conflict arbitration, and provenance‐aware integration. In this sense, the framework adds a logic for coordinating level‐specific agents and data across molecular, organelle, cellular, tissue, organ, organ‐system, and body‐system scales, rather than simply adding another task‐specific AI tool.

The conceptual distinction of the Full‐Body AI Agent lies in treating biological levels as linked reasoning contexts rather than isolated analytical domains. Outputs from one level can become structured inputs for adjacent levels, while higher‐level physiological constraints can refine or reject lower‐level explanations. For example, a molecular signal suggesting pathway disruption should not be directly converted into a clinical conclusion unless it remains consistent with cellular states, tissue organization, organ‐level physiology, and patient‐level phenotype. This bidirectional, phenotype‐oriented coordination logic defines the proposed framework. Future implementations should evaluate whether this architecture provides practical benefits over simpler alternatives using matched benchmarks, ablation‐style comparisons, and system‐level metrics such as cross‐scale alignment, arbitration success, convergence behavior, cost, latency, and failure rate.

## 4 Hierarchical Design of Collaborative Basic AI Agents for the Full‐Body AI Agent

Biological systems are inherently hierarchical, with multiple levels of organization ranging from molecular structures to cellular networks, tissues, organs, systems, and, ultimately, the entire organism. These levels interact continuously, forming a complex and dynamic network of the human body. The need for a collaborative AI Agent stems from the fact that no single AI model can effectively capture the full complexity across all biological scales. The Full‐body AI Agent must integrate a wide range of data types, including molecular profiles, cellular states, tissue properties, organ functions, and whole‐body behavior. Each of these levels requires specialized knowledge, tools, and algorithms to accurately model and predict the behavior of biological systems. To address this complexity, a collaborative design is essential. The Full‐Body AI Agent consists of multiple basic AI Agents, each dedicated to a specific level of the biological hierarchy. These agents work together, exchanging information and refining their outputs through iterative collaboration, to provide a comprehensive understanding of complex disease mechanisms. This structured hierarchy allows each basic agent to focus on its specialized domain, utilizing the most appropriate data sources and computational techniques. By distributing tasks in this way, the collaborative AI‐Agent framework ensures that the Full‐body AI Agent can synthesize a comprehensive, multi‐scale understanding of biological systems. Each basic agent focuses on its specialized biological domain while maintaining interfaces for cross‐level communication. The detailed task maps for the seven biological‐level agents are provided in Figures S1–S7. These figures use a consistent visual template to facilitate comparison across levels, but each figure represents a distinct task space corresponding to the Molecule, Organelle, Cell, Tissue, Organ, Organ System, or Body System AI Agent.

### 4.1 Illustrative Execution Trace: TP53 Mutation‐Driven Metastasis

TP53 is one of the most extensively studied tumor‐suppressor genes in cancer biology and provides a biologically suitable example for illustrating cross‐scale reasoning [74]. At the molecular level, p53 functions as a stress‐responsive transcription factor that regulates DNA‐damage response, cell‐cycle arrest, apoptosis, senescence, metabolism, and cellular survival programs [75]. In cancer, TP53 mutation can disrupt these tumor‐suppressive functions and may also produce mutant p53 gain‐of‐function activities that promote tumor progression, invasion, therapy resistance, and metastatic behavior [76]. Therefore, TP53 is not only a molecular marker, but also a useful cross‐scale example for linking gene mutation, organelle stress adaptation, altered tumor‐cell states, tissue invasion, organ‐level progression, and patient‐level metastatic phenotype.

To make the cross‐scale reasoning logic of the Full‐Body AI Agent more concrete, we use TP53 mutation‐driven metastasis as an illustrative execution trace. This example is not intended to report a runnable system or a validated agent implementation. Instead, it shows how the Full‐Body AI Agent could organize a phenotype‐oriented question into a stepwise reasoning chain in which the output of one biological level becomes the structured input for the next. The guiding question is how a TP53 alteration could propagate across biological scales and ultimately contribute to a metastatic phenotype.

At the molecular level, the Molecule AI Agent receives a TP53 molecular evidence object as input, including TP53 mutation type, mutation position, copy‐number status, transcript abundance, protein‐domain annotation, and p53 pathway context. Example data sources and tools include TCGA or cBioPortal for cancer genomic alterations, ClinVar or COSMIC for variant interpretation, Ensembl Variant Effect Predictor or ANNOVAR for variant consequence annotation, PDB or AlphaFold for protein‐structure context, and KEGG or Reactome for pathway mapping. cBioPortal supports integrative analysis of cancer genomic and clinical profiles [77], while Ensembl Variant Effect Predictor annotates variant consequences on transcripts, proteins, and regulatory regions [78]. The Molecule AI Agent converts these inputs into a TP53 molecular dysfunction object, including predicted functional consequence, affected protein domain, disrupted pathway module, confidence level, and uncertainty source. For example, a DNA‐binding‐domain mutation may support impaired p53 transcriptional regulation, defective DNA‐damage response, reduced cell‐cycle arrest, and weakened apoptotic signaling. This output does not directly predict metastasis; instead, it establishes whether TP53 dysfunction is biologically plausible as an upstream perturbation.

The TP53 molecular dysfunction object then becomes the input for the Organelle AI Agent. At the organelle level, the Organelle AI Agent integrates this molecular output with organelle‐related evidence, including mitochondrial apoptosis markers, nuclear DNA‐damage signals, oxidative‐stress indicators, mitochondrial membrane‐potential features, and genomic‐instability markers. Example tools and data types may include mitochondrial gene‐expression signatures from transcriptomic data, immunofluorescence or microscopy‐based organelle morphology analysis, CellProfiler for image‐derived organelle features, and apoptosis, oxidative‐stress, and DNA‐damage‐response pathway resources. CellProfiler provides open‐source image‐analysis workflows for quantifying cellular and subcellular phenotypes, including morphology and staining‐pattern features [79]. The Organelle AI Agent produces an organelle dysfunction object, including mitochondrial apoptosis status, nuclear stress level, oxidative‐stress adaptation, genomic‐instability evidence, confidence level, and uncertainty source. In this example, the organelle‐level output may suggest that TP53 dysfunction weakens apoptosis and allows damaged tumor cells to survive under stress.

The organelle dysfunction object is then passed to the Cell AI Agent. At the cellular level, the Cell AI Agent combines this organelle output with single‐cell or single‐nucleus transcriptomic evidence, including tumor‐cell clusters, epithelial‐to‐mesenchymal‐transition‐related gene‐expression signatures, proliferation markers, apoptosis‐resistance signals, immune‐evasion markers, and stress‐response programs. Example tools include Scanpy [80] or Seurat [81] for single‐cell or single‐nucleus RNA‐seq preprocessing, clustering, differential expression, and cell‐state annotation; Numbat [82] or SCEVAN [83] for identifying malignant epithelial cells; and gene‐set scoring methods for epithelial‐to‐mesenchymal transition, proliferation, apoptosis, and immune‐evasion signatures. The Cell AI Agent produces a cell‐state transition object, including dominant tumor‐cell state, epithelial‐to‐mesenchymal‐transition score, proliferation score, apoptosis‐resistance signal, immune‐evasion signal, confidence level, and uncertainty source. If TP53‐related organelle dysfunction is accompanied by epithelial‐to‐mesenchymal‐transition‐like programs, proliferative states, and apoptosis resistance, the Cell AI Agent interprets this as evidence that molecular and organelle‐level alterations have propagated into altered tumor‐cell behavior.

The cell‐state transition object then becomes the input for the Tissue AI Agent. At the tissue level, the Tissue AI Agent integrates this cellular output with spatial and histological evidence, including hematoxylin and eosin pathology, spatial transcriptomics, multiplex imaging, tumor‐stroma boundary annotation, immune‐cell localization, hypoxia markers, extracellular‐matrix remodeling, and vascular proximity. Example tools may include QuPath [84] for digital pathology annotation, Squidpy [85] or Giotto [86] for spatial transcriptomics analysis, SpaCCC [87] or CellPhoneDB‐like frameworks [88] for spatial cell–cell communication, and image‐analysis tools for invasion‐front or stromal‐region detection. QuPath is an open‐source platform for whole‐slide digital pathology analysis, while Squidpy provides scalable infrastructure for spatial omics analysis and tissue‐organization quantification. The Tissue AI Agent produces a tissue invasion‐context object, including invasion‐front status, location of epithelial‐to‐mesenchymal‐transition‐like tumor cells, stromal‐remodeling score, immune‐exclusion status, hypoxia evidence, vascular‐proximity evidence, confidence level, and uncertainty source. If TP53‐associated epithelial‐to‐mesenchymal‐transition‐like tumor cells are enriched at invasive margins and adjacent to remodeled stroma or immune‐excluded niches, the tissue layer supports the interpretation that cell‐state changes are spatially organized into invasion‐supportive tissue contexts.

The tissue invasion‐context object is then passed to the Organ AI Agent. At the organ level, the Organ AI Agent evaluates whether tissue‐level invasion is reflected in lung‐level anatomical or functional progression. Inputs include lesion location, tumor size, radiological imaging features, vascular invasion, pleural invasion, lymphatic proximity, bronchial involvement, and lung‐function indicators. Example tools and data sources may include computed tomography or positron emission tomography and computed tomography imaging, DICOM‐based radiomics pipelines, 3D Slicer [89] or PyRadiomics [90] for lesion and radiomic feature extraction, pathology reports for vascular or pleural invasion, and pulmonary function measurements when available. The Organ AI Agent produces an organ‐level progression object, including local invasion status, organ‐structure disruption, vascular or lymphatic access, pleural or bronchial involvement, lung‐function impact, confidence level, and uncertainty source. In this example, tissue‐level invasion near vascular or pleural structures may be converted into organ‐level evidence of aggressive local progression.

The organ‐level progression object then becomes the input for the Organ System AI Agent. At the organ‐system level, the Organ System AI Agent assesses whether lung‐level progression interacts with physiological systems involved in dissemination, including the lymphatic, circulatory, immune, respiratory, inflammatory, and coagulation systems. Inputs may include lymph‐node status, circulating tumor‐cell evidence, immune‐infiltration profiles, vascular invasion, systemic inflammatory markers, coagulation indicators, oxygenation status, and treatment‐response context. Example tools and data sources include clinical staging records, immune deconvolution tools such as CIBERSORT [91] or xCell [92], circulating tumor‐cell assays, blood biomarkers, radiological lymph‐node assessment, and clinical laboratory tests. The Organ System AI Agent produces an organ‐system dissemination object, including lymphatic involvement, vascular dissemination evidence, immune‐escape status, systemic inflammation, coagulation abnormality, respiratory‐system involvement, confidence level, and uncertainty source. This layer determines whether local lung progression is beginning to engage physiological routes of metastatic spread.

Finally, the organ‐system dissemination object is passed to the Body System AI Agent. At the body‐system level, the Body System AI Agent integrates this organ‐system output with patient‐level clinical information, including TNM stage, metastatic‐site records, recurrence status, survival data, treatment history, systemic symptoms, laboratory indicators, longitudinal imaging, and therapeutic response. Example data sources and tools may include electronic medical records, longitudinal computed tomography or positron emission tomography follow‐up, RECIST‐based response assessment [93], survival analysis, clinical risk models, and treatment‐response records. The Body System AI Agent produces a whole‐body phenotype object, including metastatic risk, current metastatic status, prognosis, treatment‐resistance likelihood, recommended validation evidence, confidence level, and uncertainty source. In this example, the body‐level output may describe TP53‐associated metastatic risk or aggressive progression only if the preceding molecular, organelle, cellular, tissue, organ, and organ‐system outputs provide coherent support.

The supervisory Full‐Body AI Agent then performs cross‐level consistency checking and physiology‐first arbitration across the seven output objects. If the outputs align, such as deleterious TP53 dysfunction, impaired apoptosis, epithelial‐to‐mesenchymal‐transition‐like tumor‐cell transition, invasion‐supportive tissue organization, lung‐level anatomical progression, dissemination‐system involvement, and clinical evidence of metastasis, the final interpretation would be cross‐scale support for TP53‐associated metastatic progression. If the outputs conflict, the framework would not force a conclusion. For example, if the Molecule AI Agent, Organelle AI Agent, and Cell AI Agent support TP53 dysfunction, impaired apoptosis, and epithelial‐to‐mesenchymal‐transition‐like tumor‐cell plasticity, but the Tissue AI Agent, Organ AI Agent, and Body System AI Agent do not support invasion, dissemination, or clinical metastasis, the final interpretation would be TP53‐associated molecular and cellular priming without a confirmed tissue‐level or systemic metastatic phenotype. The framework would then recommend additional evidence, such as spatial transcriptomics of invasive margins, pathology review, organ‐level imaging, circulating tumor‐cell assessment, or longitudinal clinical follow‐up.

This illustrative trace shows that the Full‐Body AI Agent does not explain metastasis through a direct TP53 mutation to the metastasis shortcut. Instead, each level transforms the previous level's output into a more biologically contextualized input. The Molecule AI Agent translates TP53 alteration into molecular dysfunction; the Organelle AI Agent links molecular dysfunction to apoptosis and stress adaptation; the Cell AI Agent interprets these subcellular changes as altered tumor‐cell behavior; the Tissue AI Agent tests whether these cells occupy invasion‐supportive tissue niches; the Organ AI Agent evaluates lung‐level anatomical progression; the Organ System AI Agent assesses dissemination‐related physiological systems; and the Body System AI Agent converts the accumulated evidence into a patient‐level metastatic phenotype. This stepwise structure illustrates how the Full‐Body AI Agent could provide a phenotype‐oriented framework for connecting fragmented biological evidence across scales.

### 4.2 Scalability and Modular Activation

Although the Full‐Body AI‐Agent framework defines a fine‐grained biological hierarchy spanning molecular to organismal scales, this hierarchy should be interpreted as a conceptual reasoning space rather than a set of computational layers that are simultaneously active. To maintain scalability, the framework proposes a modular activation strategy in which only a subset of biological layers and associated agents would be engaged for any given research query. Routing is guided by task context and uncertainty, allowing reasoning to begin locally and expand across scales as additional biological context becomes relevant.

In practice, most queries are expected to involve only a limited number of adjacent modules rather than the entire hierarchy. For example, a single‐cell analysis task may primarily engage molecular and cellular agents without invoking tissue‐ or organ‐level reasoning, whereas organ‐level imaging analysis may mainly involve tissue and physiological modules. Cross‐scale routing is therefore treated as a conditional escalation process, where additional layers could be activated only when intermediate uncertainty or physiological inconsistency exceeds a predefined threshold. This selective computation strategy is intended to reduce computational overhead while preserving the capacity for full cross‐scale reasoning when biologically required.

To further improve efficiency, adjacent biological layers can be conceptually organized into modular subsystems that share intermediate representations and local routing logic. Such subsystems would allow reasoning to converge locally before communicating across scales, thereby reducing unnecessary communication and synchronization costs. As a result, the framework operates conceptually as a dynamically routed hierarchy rather than a fully activated multi‐agent system, aligning computational effort with query complexity.

This design follows the principle of sparse activation commonly used in scalable expert‐based AI systems, where conditional routing can enable high representational capacity without proportional increases in computational cost [94]. By treating biological layers as selectively activated reasoning modules rather than fixed execution stages, the Full‐Body AI‐Agent framework is intended to maintain scalability while preserving biological completeness.

## 5 Agent Execution Schema, Validation, and Provenance

To enable robust integration across biological scales, basic agents in the Full‐Body AI‐Agent framework are not defined solely by abstract functional roles but are proposed to operate under standardized execution schemas. Each agent is conceptualized as a structured computational unit with explicit input–output definitions, validation checkpoints, error‐handling procedures, and provenance tracking. This design provides a consistent conceptual‐operational layer that supports reliable coordination across molecular, cellular, tissue, organ, and system‐level reasoning. To address explicit input–output interfaces, validation checkpoints, and failure handling for each basic agent, we provide a standardized execution schema and operational specification in Table S7.

Inputs to each agent would be represented through ontology‐aligned schemas that specify the biological level, representation type, measurement context, and associated uncertainty information. Inputs may include molecular features, cellular state descriptors, spatial context, or organ‐level functional constraints, depending on the role of the agent. Outputs follow similarly structured schemas containing biological predictions, confidence estimates, and transformation metadata that describe how results were derived. By enforcing schema‐constrained interfaces, cross‐agent communication becomes explicit and interpretable, reducing ambiguity during multi‐hop reasoning and enabling consistent routing between biological layers.

Validation gates are proposed between execution stages to ensure that outputs satisfy schema consistency and biological plausibility before propagation. These checkpoints would verify ontology compliance, compatibility with higher‐level physiological constraints, and coherence with upstream evidence. Outputs that fail validation or exhibit insufficient confidence would not be propagated directly; instead, they would trigger controlled error‐handling procedures such as refinement with additional context, rerouting to alternative agents, or escalation to neighboring biological layers. Such mechanisms are intended to prevent unstable intermediate results from cascading through the reasoning pipeline.

To support reproducibility and traceability, every execution step would generate provenance records describing inputs, invoked tools, transformation operations, model versions, and uncertainty updates. These records would form a traceable reasoning graph that allows reconstruction and auditing of multi‐hop inference chains and facilitates localization of error sources when inconsistencies arise. Through standardized execution schemas, validation checkpoints, explicit error handling, and provenance tracking, the Full‐Body AI‐Agent framework provides a structured and auditable basis for agent‐level reasoning, supporting safer and more reliable cross‐scale biological inference.

## 6 Applications of the Full‐Body AI‐Agent System: Case Studies

**FIGURE 4:**
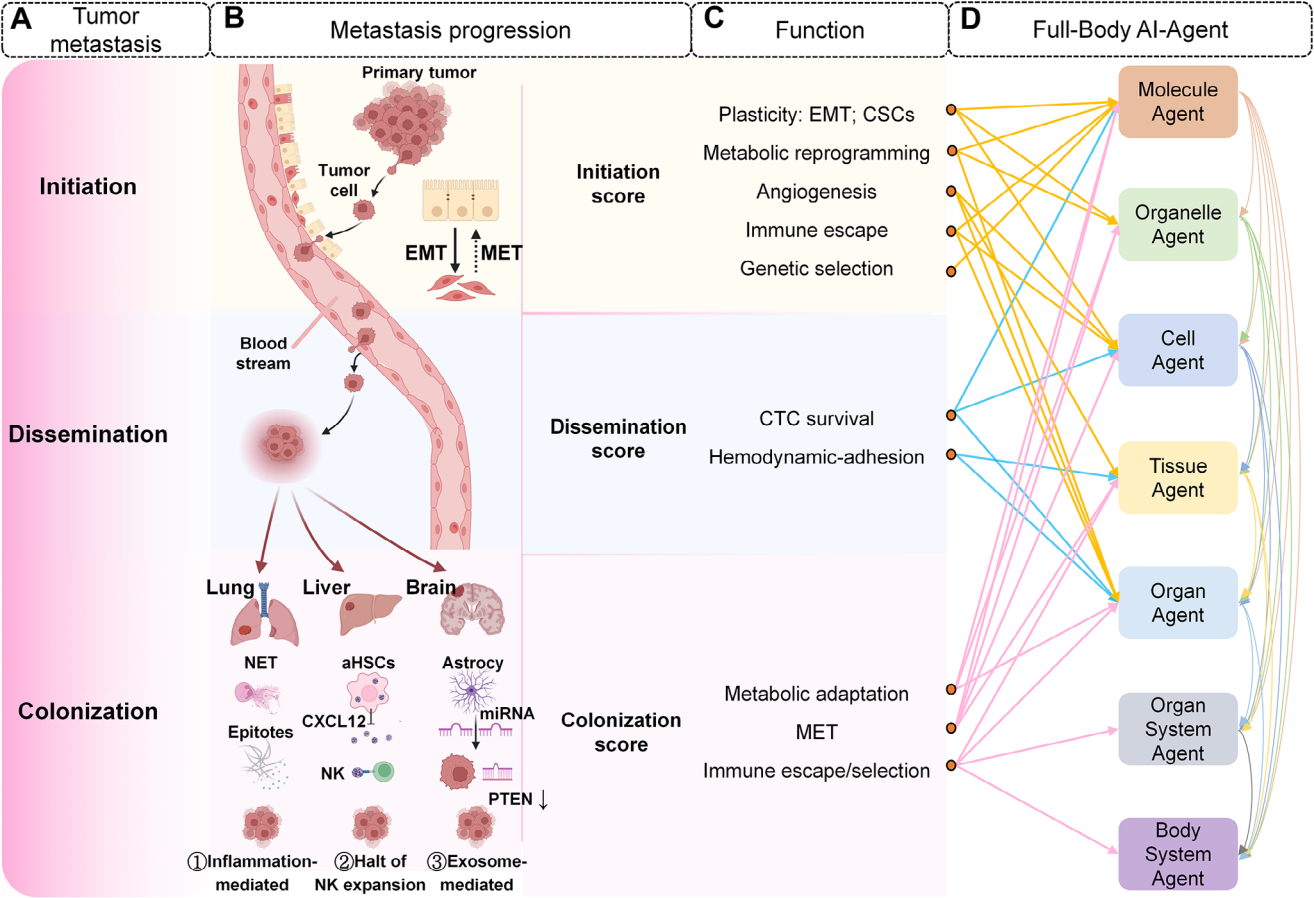
Metastasis AI Agent, a three‐phase metastasis scoring framework leveraging the Full‐Body AI‐Agent system. (A) The metastatic cascade unfolds in three phases: primary tumor invasion, vascular dissemination, and distant colonization. (B) Organ‐specific colonization mechanisms: lung trapping by NETs/CXCL12, liver dormancy enforced by stellate cell‐mediated NK suppression, and brain outgrowth primed by astrocyte exosomal PTEN loss. (C) Quantitative scoring of each phase (Initiation, Dissemination, and Colonization) captures the key biological hurdles. (D) Integration of multi‐scale, multi‐modal features, from molecular to systemic, enables comprehensive evaluation of metastatic potential.

**FIGURE 5:**
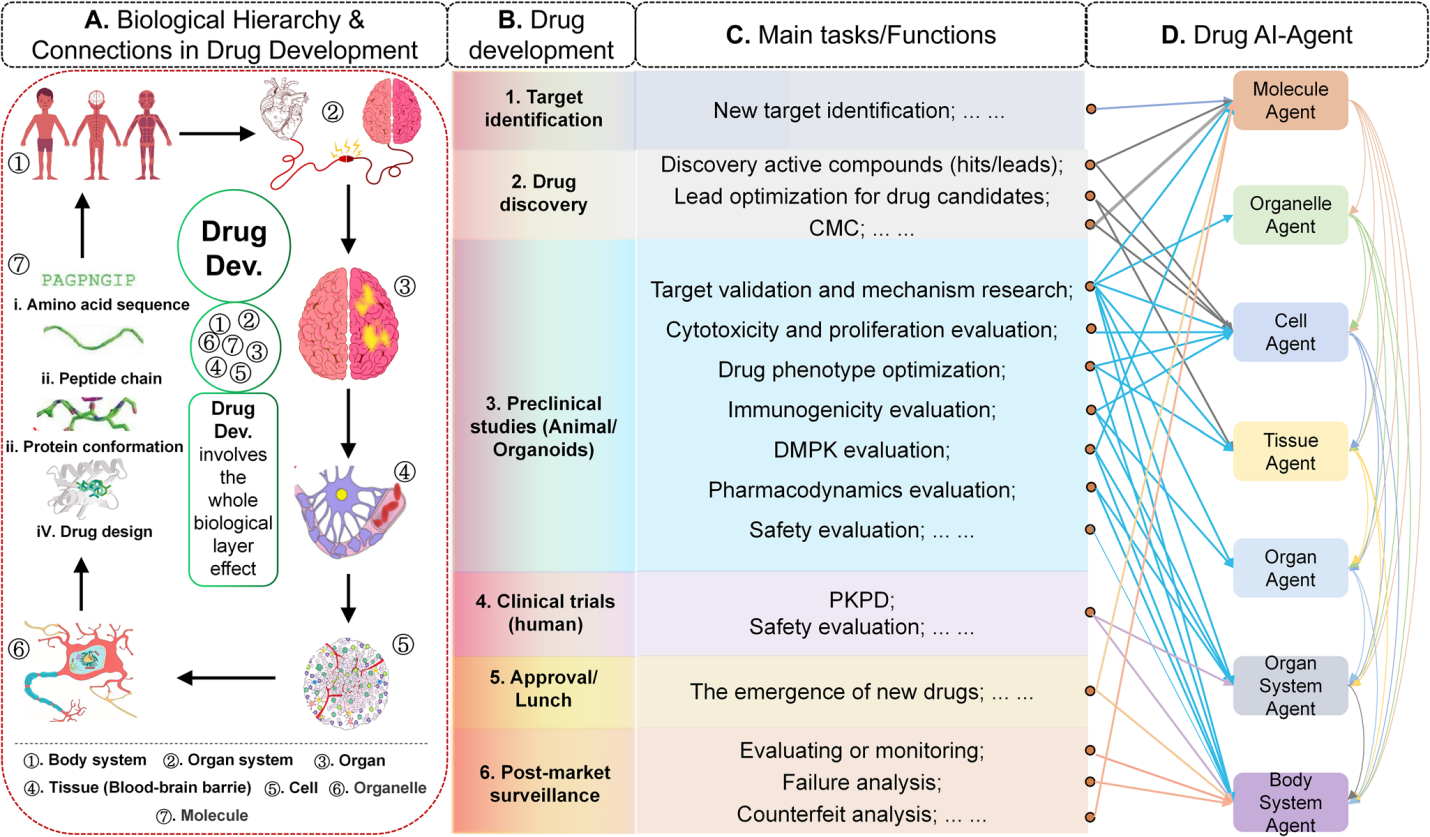
Integration of biological hierarchy, drug development workflow, and drug AI‐Agent architecture. This figure illustrates the multi‐scale integration of biological organization (A) into the canonical drug development pipeline (B). Panel (A) depicts the seven interconnected biological scales (molecule, organelle, cell, tissue, organ, organ system, and body system) that underpin drug action, emphasizing that drug development inherently involves effects and considerations spanning these entire layers. Panel (B) maps these biological scales to the sequential stages of drug development—from target identification, drug discovery, and preclinical studies (including the use of organoids and animal models) to clinical trials, approval, and post‐market surveillance—highlighting how each stage relies on insights from specific biological levels. Panel (C) outlines the major tasks and biological functions required across development stages, while Panel (D) shows how each foundational AI‐Agent within the Full‐Body AI‐Agent framework contributes to these tasks. The Drug AI‐Agent functions as an integrative layer that synthesizes outputs across all levels to support system‐wide prediction, optimization, and feedback in drug development.

The Full‐body AI‐Agent framework is proposed to support a systematic, multi‐scale approach to dissecting disease mechanisms and optimizing therapeutic interventions. Here, we present two case studies: the exploration of Lung cancer metastasis (Figure 4) and Drug development (Figure 5), demonstrating the framework's potential in both disease research and clinical translation.

### 6.1 Case 1: A Three‐Phase Metastasis Scoring Framework Leveraging Full‐Body AI‐Agents

Metastasis, the spread of malignant cells from a primary tumor to distant organs, is responsible for the majority of cancer‐related deaths. It is widely conceptualized as a series of sequential events, termed the invasion‐metastasis cascade [95]. Tumor metastasis unfolds in three successive phases (Figure 4A). Figure 4B shows the occurrence and progression of tumor metastasis. In the Initiation phase, epithelial tumor cells undergo epithelial‐to‐mesenchymal transition (EMT) to gain motility and invasive potential [96]; then, in coordination with cancer stem‐cell traits [97], metabolic reprogramming [98], angiogenic remodeling [99], immune evasion [100], and clonal selection [101] to breach the basement membrane and intravasate. During the Dissemination phase, circulating tumor cells (CTCs) face multiple survival challenges, including hemodynamic shear stress, anoikis, and immune attack. To overcome these barriers, CTCs employ mechanisms such as platelet cloaking and endothelial adhesion, allowing them to traverse the vasculature [102]. Finally, in the Colonization phase, disseminated tumor cells extravasate into distant tissues and often revert to an epithelial phenotype through mesenchymal‐to‐epithelial transition (MET) [103]. These cells may enter a dormant state and, under the influence of late‐stage immune suppression and supportive microenvironmental cues, become reactivated to form overt macroscopic metastases. Importantly, tumor colonization at distant sites is governed by organ‐specific microenvironmental signals that uniquely shape the trajectory of metastatic outgrowth [104]. For instance, in the lung, circulating tumor cells are entrapped and activated by neutrophil extracellular traps (NETs) and endothelial *CXCL12* gradients, which cooperate to awaken dormant cells and promote early micrometastatic seeding [105]. In the liver, activated hepatic stellate cells impose a fibrotic niche that suppresses natural killer cell‐mediated clearance, enforcing prolonged dormancy of disseminated tumor cells until niche remodeling permits their reawakening [106]. In the brain, astrocyte‐derived exosomal microRNAs induce *PTEN* downregulation in tumor cells, creating a growth‐permissive niche that primes metastatic cells for proliferation [107]. These examples illustrate how each organ's unique stromal and immune landscapes dictate the survival, dormancy, and eventual outgrowth of disseminated cancer cells. The complexity and organ‐specific nature of metastasis underscore the need for a global perspective that integrates multimodal data and mechanistic insights across molecular, cellular, tissue, organ, and systemic scales to fully elucidate its dynamic progression and interdependencies.

Integrating across all biological scales is essential to understanding tumor metastasis. Despite significant advances at individual biological scales, from gene discovery to organ‐level imaging, a comprehensive understanding of how molecular events cascade through cells, tissues, and organs to manifest as systemic diseases remains elusive. To bridge this gap, we introduce the Full‐Body AI‐Agent, a unified framework composed of seven foundational AI modules, each corresponding to a specific level of biological organization. Based on multi‐omics and multi‐modal data, and by integrating insights from molecules, organelles, cells, tissues, organs, inter‐organ interactions, and full‐body physiology, this framework enables the reconstruction of complex biological networks with unprecedented resolution and interpretability. The Full‐Body AI Agent is not intended to include every AI analysis that integrates two or more biological levels. Instead, it refers to a phenotype‐oriented coordination framework in which level‐specific data and tools are connected through biological‐layer routing, bidirectional constraints, uncertainty tracking, and traceable provenance. The Metastasis AI Agent serves as a prototypical example to model and decode the multi‐step, multi‐organ nature of cancer metastasis.

We anchored our Metastasis AI Agent on three distinct yet interdependent metrics: the Initiation score, Dissemination score, and Colonization score (Figure 4C). These scores correspond to the fundamental biological challenges that tumor cells must overcome during their progression from the primary site to distant metastatic sites. The Initiation score assesses a tumor's ability to detach from the primary site, invade surrounding tissues, and evade local immune surveillance. The Dissemination score quantifies the capacity of CTCs to survive hemodynamic stress, resist immune attack, and adhere to the endothelium at distant sites. The Colonization score evaluates the likelihood that disseminated tumor cells will enter a dormant state, adapt to a new niche, and ultimately resume proliferation to form overt metastases. By focusing on these three scores, the Metastasis AI Agent pinpoints the specific vulnerabilities at each stage, guides targeted data integration across biological scales, and enables phase‐specific risk assessment and therapeutic intervention. Here, we detail how Metastasis AI Agent achieves a Full‐Body understanding of Metastasis through the basic AI Agent at seven biological levels and cross‐scale quantification of metastatic potential via three scores (Figure 4D).

To evaluate the Initiation core, the Molecule AI Agent first quantifies the expression of EMT drivers (such as *SNAIL* and *TWIST*), stemness regulators (including *NANOG* and *SOX2*), and genetic alterations that confer a selective advantage to tumor cells. Next, the Cell AI Agent analyzes single‐cell phenotypes to gauge the prevalence of hybrid EMT states, cancer stem cell‐like clusters, and shifts in metabolic pathway activity indicative of glycolytic reprogramming versus oxidative phosphorylation. Concurrently, the Tissue AI Agent evaluates angiogenic remodeling by quantifying microvessel density and perivascular cell infiltration from segmented immunohistochemistry data. The Organ AI Agent extracts metrics of perfusion heterogeneity and vessel permeability metrics from multiparametric imaging. In parallel, the Cell and Tissue AI‐Agents collaboratively score local immune escape by identifying *PD‐L1* expression patterns and the presence of immunosuppressive cell populations. Combined through a multilayer ensemble, these heterogeneous features yield a continuous Initiation Score that could help stratify tumors based on their early invasive potential.

The Dissemination Score assesses the ability of circulating tumor cells to survive hemodynamic shear stress, resist anoikis, and adhere within distant vasculature. The Cell AI Agent profiles CTCs to identify transcriptional programs that promote anchorage‐independent survival, including anoikis resistance and upregulation of platelet‐interacting receptors. Meanwhile, the Organ‐System AI Agent constructs patient‐specific hemodynamic simulations from four‐dimensional flow MRI and computational fluid dynamics, pinpointing vascular regions of low shear stress that favor tumor cell arrest and platelet cloaking. The Tissue AI Agent quantifies endothelial adhesion molecule expression, such as *ICAM‐1* and *VCAM‐1*, through spatial transcriptomics data to map vascular adhesion hotspots. By integrating intrinsic survival signatures with organ‐axis fluid dynamics and adhesion landscapes, the Dissemination Score captures a cell's capacity to transit, persist, and arrest within the circulatory system.

To assess the Colonization Score, the Molecule AI‐Agent examines single‐cell metabolic profiles of dormant cells to detect shifts toward fatty‐acid oxidation and enhanced antioxidant pathways. The Cell AI‐Agent assesses the extent of MET by measuring the re‐expression of epithelial markers and the restoration of junctional complexes. The Tissue AI‐Agent characterizes the extracellular matrix by profiling its composition, stiffness gradients, and vascular density through multiplexed imaging to define niche permissiveness. The Organ AI‐Agent integrates PET (Positron Emission Tomography) tracer uptake and metabolomics data to map organ‐specific nutrient availability, while the Organ‐System AI‐Agent models cross‐organ immune‐suppression axes, such as the recruitment of bone marrow‐derived myeloid cells, to capture late‐stage immune editing. By harmonizing these multi‐scale features, the Colonization Score could estimate the likelihood of dormant cell survival, niche integration, and metastatic outgrowth.

To provide a concrete illustration of how metastatic potential could be organized during the Initiation phase, we describe a representative snRNA‐seq‐based workflow. This example is intended to show how molecular and cellular features could be structured within the Initiation module, rather than to report a validated score or an implemented Metastasis AI Agent. In a future implementation, the Initiation module could integrate molecular, cellular, tissue, and organ‐level features, with each feature type extracted or interpreted by the corresponding biological‐level AI Agent.

At the molecular level, the Molecular AI Agent would incorporate epithelial‐to‐mesenchymal transition drivers, cancer stemness regulators, metabolic reprogramming signatures, and genetic alterations that may confer selective growth advantages. For snRNA‐seq input, preprocessing could be performed using Scanpy, including quality control, normalization, log‐transformation, highly variable gene selection, dimensionality reduction, clustering, and downstream analysis of malignant epithelial cells. Marker gene sets representing epithelial and mesenchymal states, EMT drivers, stemness regulators, glycolysis, and oxidative phosphorylation could then be curated. Using gene‐set scoring methods, per‐cell epithelial, mesenchymal, stemness, glycolytic, and oxidative phosphorylation scores could be estimated.

A hybrid EMT score could be defined by the overlap or co‐activation of epithelial and mesenchymal programs, while a metabolic reprogramming score could represent the relative balance between glycolysis and oxidative phosphorylation. These features could be normalized and combined into an illustrative molecular initiation profile. At the cellular level, the Cell AI Agent would evaluate the prevalence of hybrid EMT phenotypes, cancer stem cell‐like states, proliferative clusters, and apoptosis‐resistant or stress‐adapted malignant‐cell populations. Clustering malignant cells, for example, using Louvain or related graph‐based methods, could help map cellular subpopulations against hybrid EMT and stemness scores [108].

Tissue‐ and organ‐level evidence would require additional data modalities beyond snRNA‐seq. The Tissue AI Agent would require histopathology, spatial transcriptomics, multiplex imaging, or tumor‐microenvironment annotations to evaluate angiogenic remodeling, microvessel density, stromal remodeling, immune exclusion, and local invasion. The Organ AI Agent would require multiparametric imaging, radiomics, vascular invasion assessment, perfusion heterogeneity, or organ‐function indicators to determine whether local tissue invasion is reflected in organ‐level progression.

This example shows how molecular and cellular features could be prepared for future integration with tissue‐, organ‐, organ‐system‐, and body‐level evidence. Representative output formats for the Initiation, Dissemination, and Colonization modules are provided in Table S8 as conceptual examples.

Cross‐scale metastasis reasoning may fail when evidence from different biological levels points in different directions. For example, molecular or cellular features may suggest high metastatic potential, such as EMT‐like transcriptional programs, stemness signatures, or apoptosis resistance, whereas tissue‐ or organ‐level evidence may not support invasion, vascular access, or systemic dissemination. In such cases, the Metastasis AI Agent should not force a high‐risk conclusion from lower‐level signals alone. Instead, the supervisory Full‐Body AI Agent would flag a cross‐layer conflict, retain alternative interpretations, and downgrade the confidence of the metastasis module until additional spatial, pathological, imaging, or longitudinal clinical evidence becomes available. Thus, a tumor may be interpreted as showing molecular or cellular priming for metastasis without confirmed tissue‐level invasion or body‐level metastatic phenotype.

### 6.2 Case 2: The Application of the Full‐Body AI Agent Platform in Drug Development

The enduring human aspiration for health and longevity continues to drive scientific efforts in drug development. Yet, the complexity of the human body remains only partially understood, presenting significant obstacles to the design, evaluation, and deployment of effective therapies. In Figure 5A, drug development is conceptualized as a biological staircase, one that must be traversed across multiple interconnected levels of biological organization. This multi‐scale perspective highlights the importance of integrating molecular mechanisms with systemic physiological responses. It illustrates how each layer, from protein structures to whole‐body dynamics, contributes to the success or failure of a therapeutic strategy. At the whole‐body system level (1), drug effects manifest as changes in global neuroendocrine, immune, and behavioral states. These systemic responses are shaped by the integrated dynamics of multiple organ systems and reflect the ultimate therapeutic outcome, which can be either an improvement or an adverse reaction. This level defines the clinical endpoints by which treatment efficacy is judged, including survival, symptom relief, or improvement in quality of life, thus anchoring the top of the biological staircase. Descending to the organ system level (2), interorgan communication becomes central. A notable example is the heart–brain axis, where physiological signals such as cardiac rhythm and cerebrovascular pressure allow the heart to influence brain function [109]. Such interactions underscore the importance of accounting for multi‐organ crosstalk during drug development. At the organ level (3), anatomical structures become the focus point. Here, the brain is shown with a localized tumor, representing a pathological region embedded within a complex functional organ. The structural localization of disease introduces challenges in spatial drug delivery and highlights the need for targeting strategies that consider both regional anatomy and functional compartmentalization. The tissue and microenvironment level (4) provides a more granular perspective, focusing on the tumor microenvironment, including the tumor surrounding matrix, immune context, and vasculature, notably the blood–brain barrier (BBB). The BBB represents a formidable obstacle to drug permeability, representing both a physical and biological barrier that must be overcome to achieve adequate drug concentrations at the site of action. At the cellular level (5), the tumor is no longer viewed as a bulk mass but rather as a heterogeneous population of individual cells, each exhibiting distinct transcriptional profiles, phenotypic behaviors, and drug sensitivities. Achieving single‐cell resolution is essential for uncovering drug resistance, clonal evolution, and cell type‐specific responses that are often masked in bulk analyses.

Descending further, the subcellular level (6) focuses on intracellular alterations such as organelle dysfunction, disrupted signaling pathways, and metabolic stress, which collectively shape how a cell perceives and responds to a therapeutic agent. This level captures early mechanistic signals that link molecular intervention to phenotypic change, offering critical insights into both drug efficacy and toxicity. At the base of the staircase lies the molecular level (7), where drug‐target interactions are determined by atomic‐scale features, such as protein conformation, binding pocket geometry, and ligand specificity. It is at this foundational level that pharmacological action originates and where medicinal chemistry plays a central role in optimizing structure–activity relationships to initiate therapeutic cascades.

As shown in Figure 5A, the Full‐Body AI‐Agent framework introduces a phenotypic‐guided approach to drug development, where therapeutic interventions are continuously optimized based on their impact on human phenotypes. This process forms a closed‐loop cycle: the drug development process begins by focusing on the phenotypic outcomes of interest, and as the drug design evolves, it aims to modulate those phenotypes at the molecular and cellular levels. As the drug acts on molecular targets, it induces physiological changes that are then monitored and used to refine the drug's design. This feedback loop ensures that drug candidates are directly aligned with the desired clinical outcomes, making drug development more biologically relevant and efficient. In contrast, recent models like AlphaGenome [110] and Evo2 [39] excel in molecular‐level mechanistic understanding but lack direct mapping to phenotypic responses. While these models provide valuable insights into genetic and protein‐level dynamics, they fall short of integrating these molecular insights into a broader phenotypic context, thus limiting their applicability to real‐world biological problems. The Full‐Body AI‐Agent framework, by emphasizing phenotype‐driven drug development, shifts the focus back to the human organism and its clinical relevance, enabling a more integrated and holistic approach. This framework fosters a deeper connection between molecular mechanisms and observable human phenotypes, ensuring that therapeutic strategies are aligned with their ultimate clinical objectives from the outset.

Drug development strategies have expanded significantly in recent years, yet clinical translation remains a persistent bottleneck. In practice, more than 90% of drug candidates deemed “safe and effective” in animal models ultimately fail in human clinical trials, primarily due to insufficient efficacy or unacceptable toxicity [111, 112]. This high attrition highlights the inherent limitations of animal models in predicting human outcomes. Recognizing this, the U.S. Food and Drug Administration (FDA), through its Reducing Animal Testing Roadmap, has identified interspecies immunogenicity and physiological divergence as the key contributors to this disconnection. As a result, the FDA has formally endorsed the development of human‐relevant platforms such as organoids to reduce reliance on animal studies and enhance translational success.

Organoids and organ‐on‐a‐chip systems, which combine three‐dimensional human cell self‐assembly with microfluidic biomimicry, offer high‐fidelity recapitulation of localized organ function [113]. A notable milestone was achieved when a lung‐on‐chip model enabled Azeliragon to advance directly to Phase II clinical trials, demonstrating the potential of such systems to accelerate the transition from in vitro discovery to human validation [114]. However, as these technologies advance, their limitations must also be critically examined from a systems‐level perspective. Two fundamental constraints are particularly salient. First is the boundedness of biological systems: the human body is a closed, dynamically coupled network of interacting organs. Most current chip models focus on single organs or limited pairings, making it difficult to recapitulate the cascading metabolism and distal toxicities that unfold along the liver–kidney–gut–immune axis. Second is the orderliness of biological processes: many chronic toxicities emerge only after weeks or months of accumulation and systemic feedback. Yet, the duration and stability of current chip cultures rarely support such long‐term observation windows. Recent reviews have also noted structural limitations. For example, even advanced liver organoids often lack critical cell populations such as cholangiocytes and vascular endothelial cells, which impairs their ability to model drug bioactivation and metabolic toxicity with sufficient accuracy [115, 116]. New strategies for preclinical screening are urgently needed to better predict clinical outcomes, thereby expediting drug development while maintaining cost‐effectiveness.

To systematically address the limitations of conventional preclinical models and enhance the fidelity of translational research, we introduce the Drug AI‐Agent, a core module of the Full‐Body AI‐Agent framework, as illustrated in Figure 5. Figure 5A, Wang, H., Fu, T., Du, Y. et al. Scientific discovery in the age of artificial intelligence. A outlines the full spectrum of biological hierarchy and its integration into drug development, spanning from molecular structures to systemic physiological responses. Drug development proceeds through six canonical stages (Figure 5B), beginning with target identification and compound discovery, progressing through preclinical validation, and culminating in clinical trials, regulatory approval, and post‐market surveillance [117]. Within this workflow, organoids and organ‐on‐chip models are primarily positioned in Step 3, serving as key platforms for preclinical evaluation of safety, efficacy, and pharmacodynamics. Figure 5C highlights a broad set of functional requirements that span all stages of drug development, from target validation and immunogenicity screening to pharmacokinetics, toxicity assessment, and post‐market failure analysis. Meeting these demands requires scalable, human‐relevant, and biologically contextualized modeling across multiple layers of biological organization.

Within the Full‐Body AI‐Agent framework, the Drug AI‐Agent serves as a higher‐order integrative module that synthesizes insights from seven foundational AI‐Agents to enable comprehensive, system‐aware drug development. Each foundational agent leverages level‐specific biomedical data to fulfill key tasks. These agents collectively simulate and analyze the whole cascade of drug action. This architecture allows insights generated at the molecular or cellular level to propagate upward through tissues, organs, and entire physiological systems. Conversely, systemic constraints such as inter‐organ signaling or immune response feedback downward to inform drug design, delivery, and dosing strategies. In this way, the Drug AI‐Agent provides a computationally integrated, causally aware, and physiologically grounded alternative to conventional preclinical workflows, embedding organoid‐derived findings into a dynamic whole‐body context.

Figure 5D shows the biological levels involved in various tasks and biological functions during the drug development process, demonstrating the complexity of multiple biological levels in the drug development process. By aggregating outputs from all these levels, the Drug AI‐Agent constructs a dynamic, multi‐scale understanding of how molecular interventions propagate through cellular, tissue, and organ systems to shape organism‐level responses. This unified view is intended to support future efficacy assessment, systemic toxicity forecasting, and design feedback loops, bridging the gap between in vitro models and in vivo clinical outcomes.

Building on the structural and functional insights described above, the Full Body AI‐Agent could, in principle, support components of an end‐to‐end in‐silico drug discovery workflow. First, in‐house and public compound libraries are virtually screened against predicted binding pockets using high‐throughput docking tools such as AutoDock‐GPU [118] and GNINA [119] to generate binding‐affinity scores. Promising hits are refined through molecular dynamics simulations (e.g., GROMACS [120]) and free‐energy perturbation analyses to optimize binding poses and estimate binding free energyΔG). The agent then invokes generative models such as DeepChain [121], DiffDock [122], and equivariant diffusion networks to de‐novo design analogs optimized for multiple objectives, including potency, selectivity, synthesizability (SA score), and patentability. Each candidate is evaluated through rapid ADMET (Absorption, Distribution, Metabolism, Excretion, and Toxicity) prediction modules: pkCSM [123] for pharmacokinetics, hERG‐Block for cardiac safety, and Tox21 ensemble models for general toxicity profiling. Finally, synthesis routes are proposed via retrosynthetic planning (RETRO models [124], ASKCOS [125]) and prioritized based on cost and synthetic step count. These in‐silico outputs feed into experimental loops (biochemical assay optimization, SPR/ITC binding validation) that iteratively retrain the agent, effectively closing the design‐make‐test‐analyze cycle.

Recent real‐world applications illustrate the transformative potential of AI‐agent‐driven reasoning in drug development: ISM001‐055 (INS018_055), developed by Insilico Medicine, is the first AI‐discovered and AI‐designed drug candidate for idiopathic pulmonary fibrosis (IPF), identified via generative AI for novel target discovery, now successfully completing Phase IIa trials with dose‐dependent lung function improvement, good safety, and FDA orphan drug designation [126]; Halicin, discovered at MIT through AI‐based antibiotic screening, represents a novel broad‐spectrum antibiotic effective against multiple drug‐resistant pathogens, which eradicated otherwise untreatable infections in murine models and exemplifies AI's ability to explore novel chemical spaces despite still being in preclinical stages [127]; Baricitinib, identified by BenevolentAI's platform, was repurposed from rheumatoid arthritis treatment to target SARS‐CoV‐2‐related inflammation, progressing from AI‐generated hypothesis to FDA emergency use authorization in just nine months, demonstrating unprecedented acceleration in translational timelines [128]. Collectively, these cases underscore that when multi‐agent architectures are applied, especially those with cross‐scale integration like the Full‐Body AI‐Agent, the drug development process can transcend conventional siloed stages. By unifying molecular discovery, organoid modeling, and whole‐body simulation within a single reasoning framework, such systems may help address translational gaps in future validated implementations and enable faster, more cost‐effective delivery of safe and effective therapies.

The Drug AI Agent may also encounter conflicts when localized preclinical models and whole‐body constraints provide inconsistent evidence. For example, an organoid or organ‐on‐chip model may show favorable tumor‐cell killing or target engagement, while whole‐body reasoning may indicate poor pharmacokinetics, immune‐mediated toxicity, liver or kidney vulnerability, blood–brain barrier limitations, or adverse inter‐organ feedback. In this situation, the Drug AI Agent should not directly extrapolate local efficacy to systemic therapeutic success. Instead, the supervisory Full‐Body AI Agent would flag the organoid‐to‐body mismatch, reduce confidence in the candidate, and request additional evidence, such as multi‐organ chip validation, pharmacokinetic and pharmacodynamic modeling, toxicity screening, immune‐response assessment, or longitudinal animal and clinical data. This conflict‐aware design is intended to prevent local model success from being overinterpreted as whole‐body therapeutic efficacy.

## 7 Current Progress and Challenges

This Perspective does not report a completed, runnable, or benchmarked Full‐Body AI Agent software platform. Instead, it proposes a conceptual and operational blueprint for organizing future biology‐constrained multi‐agent systems. The supervisory Full‐Body AI Agent, the seven biological‐level agents, cross‐scale routing logic, message schemas, validation gates, physiology‐first arbitration rules, uncertainty propagation, external tool orchestration, and provenance tracking are described as conceptual or protocol‐level design components rather than deployed software modules. Existing biomedical agents and computational tools discussed in this manuscript are presented as examples of systems that could be positioned within such a framework in future implementations, not as components already integrated into a single runnable platform. The case studies are illustrative scenarios designed to clarify how fragmented biological evidence could be organized across scales; they are not presented as outputs from implemented agents or as validated predictive systems.

By integrating artificial intelligence across multiple biological scales, the Full‐Body AI Agent framework represents a paradigm shift in how human biology is modeled and interpreted. While recent advances demonstrate its promise, several key challenges remain. These challenges encompass existing data availability, technical limitations, and ethical considerations. Addressing these obstacles is crucial to realizing the full potential of this integrative, multi‐scale approach to biomedical research and clinical application.

### 7.1 Existing Data and Technical Support

Despite significant advancements in computational biology and AI, several technical bottlenecks must be addressed to fully realize the potential of the Full‐Body AI‐Agent framework. A major challenge is integrating heterogeneous data. Biological data comes from diverse sources, including genomic sequences, proteomic profiles, clinical records, and imaging data. These data types often differ in format, resolution, and scale, making it difficult to integrate them seamlessly into a unified model. In addition, cross‐scale data integration is further complicated by differences in sampling frequency, measurement uncertainty, and noise structure across biological levels (e.g., dropout effects in single‐cell data, resolution limitations in spatial omics, and coarse‐grained representations in organ‐level imaging) [50, 129, 130]. As a result, harmonization across scales cannot fully preserve all biological information and necessarily requires intermediate abstractions and uncertainty‐aware integration strategies. The challenge of standardizing and harmonizing these datasets is a critical hurdle for the framework.

Another challenge is model interpretability. Deep learning models, particularly those used in complex biological simulations, often function as “black boxes,” meaning it is difficult to understand how they generate predictions or insights. In biological research, it is essential that the model outputs be interpretable so that they can be validated, trusted, and used effectively. This calls for the development of explainable AI (XAI) techniques that can shed light on how models arrive at their conclusions, especially when these insights will inform clinical decisions or influence therapeutic strategies.

Scalability is also a significant issue. Modeling human biology across multiple levels requires immense computational resources. Simulating the dynamic interactions between these layers in real time involves handling massive datasets and complex algorithms. Current computational models may not be sufficient to scale with the increasing complexity of biological systems. Developing more efficient and scalable algorithms will be necessary to overcome this barrier and ensure that the Full‐Body AI‐Agent can handle the high computational demands of multilevel biological simulations. Moreover, the operational logic of the agent framework introduces additional complexity, since stable reasoning requires structured coordination, conditional model routing of agents, and validation checkpoints to prevent error propagation during multi‐step cross‐scale inference [131, 132].

Furthermore, data quality and noise are ongoing challenges. Biological datasets are often incomplete or noisy, due to sampling biases, technical limitations, or variations between experiments. The Full‐Body AI‐Agent will need to incorporate robust methods for cleaning and standardizing data to minimize these issues. Missing values, inconsistencies, and noise must be addressed to ensure that the model is built on high‐quality data, which is crucial for producing accurate and reliable predictions. Finally, the refinement of computational models and algorithms is an ongoing process. Biological systems are highly dynamic and complex, often exhibiting nonlinear relationships and feedback loops. Current AI models may not be able to accurately capture the full complexity of these systems, so there is a need for continuous improvement in the algorithms used by the Full‐Body AI‐Agent to better simulate these intricate biological processes.

### 7.2 Technical Bottleneck and Extensions

Currently, there is a significant lack of effective tools capable of characterizing the human body across all biological levels, especially at the levels of organ interactions and whole‐body systems. To address these gaps, we have developed and proposed specialized methods and tools aiming at enhancing the capabilities of the Full‐body AI Agent in future applications. One such innovation is Spatial‐GWAS, a method we defined to enable a more comprehensive investigation of human systemic biology integrated into the Full‐Body AI‐Agent framework, which simultaneously considers brain spatial interactions and genetic association studies, improving the accuracy of identifying important genetic variations and elucidating their interactions.

### 7.3 Ethics and Application Limitations

The Full‐Body AI‐Agent framework raises several ethical concerns, primarily around data privacy and security. Protecting sensitive patient data, including genomic and clinical information, requires robust encryption, anonymization, and secure access protocols to ensure confidentiality and maintain patient privacy. Bias and fairness are also significant issues, as unrepresentative training data could lead to biased predictions and health disparities. Additionally, the framework must undergo clinical validation to ensure accuracy and reliability before being used in healthcare settings. Transparency and accountability are key, as AI‐generated insights must complement human expertise with clear guidelines on decision‐making responsibilities. Finally, effective regulatory oversight will be crucial to ensuring compliance with healthcare standards and ethical practices in real‐world applications.

## 8 Discussion

The Full‐Body AI‐Agent framework, as a conceptual and operational blueprint, presents an innovative approach to organizing cross‐scale biological reasoning through a network of specialized AI Agents. Rather than presenting a completed software platform, this Perspective describes how molecular, organelle, cellular, tissue, organ, organ‐system, and body‐system evidence could be linked through phenotype‐oriented task decomposition, bidirectional routing, physiology‐first arbitration, and traceable provenance. This framework envisions the coordinated organization of diverse biological data sources, including genetic data, chemical and physicochemical properties, cell biology data, physiological data, anatomical structures, and multi‐scale imaging. By unifying these data resources, the Full‐Body AI Agent may help expand the scope and resolution of biomedical research in future implementations. Unlike domain‐bounded systems that optimize for isolated workflow segments, the Full‐Body AI‐Agent is proposed to organize reasoning as a dynamic, cross‐scale process. This shift has implications beyond mere technical integration: it reframes how computational systems could support the interpretation of biological causality, not by aggregating static outputs, but by allowing hypotheses to evolve through iterative, bidirectional exchanges between mechanistic and systemic levels.

The main contribution of this Perspective is therefore not a new biological discovery or a validated predictive system, but a biology‐constrained coordination abstraction for connecting fragmented biomedical data, tools, and interpretations across scales. Methodologically, the framework addresses two enduring weaknesses in biomedical AI. First, it aims to reduce the fidelity loss that occurs when molecular‐ or organ‐level findings are extrapolated without systemic context, by requiring lower‐level outputs to be evaluated against adjacent or higher‐level evidence and constraints. Second, it expands inference from targeted problem solving to phenotype‐oriented hypothesis generation, enabling the future analysis of emergent properties, inter‐organ interactions, and polypharmacological effects, phenomena that are often inaccessible to reductionist approaches.

A key potential utility lies in its ability to situate experimental platforms such as organoids, organ‐on‐chip systems, and in vivo studies within a coherent computational reasoning loop. Rather than displacing these methods, the Full‐Body AI‐Agent could help prioritize data acquisition, identify high‐value experimental validations, and reduce redundant testing in future implementations. This coupling of in silico and wet‐lab pipelines may help address persistent bottlenecks and high attrition rates in drug development, a challenge well‐documented in both academic and industrial settings.

Realizing this potential, however, requires overcoming substantial technical barriers. Data fragmentation remains a critical constraint: biological datasets vary widely in resolution, scale, and modality, impeding seamless integration. Standardization and harmonization pipelines, for both experimental outputs and public databases, will be essential for enabling multi‐agent collaboration. Furthermore, the temporal alignment of heterogeneous datasets, particularly for dynamic physiological processes, is still a largely unsolved problem. On the computational side, organism‐scale simulations with sufficient fidelity remain resource‐intensive, and interpretability of multi‐scale causal chains remains a major barrier to clinical adoption.

Advances in experimental biology will play a decisive role. High‐resolution, multidimensional modalities such as single‐cell and spatial omics, multi‐scale imaging, and continuous physiological monitoring are essential to populate the framework's reasoning layers with relevant, time‐resolved data. Parallel developments in AI, including deep learning, reinforcement learning, and LLMs, will further enhance the system's capacity to process diverse data types and integrate structured and unstructured biomedical knowledge. Yet, the “black box” nature of many deep models poses challenges for explainability. Building trust, particularly in clinical settings, will require interpretability mechanisms that transparently map AI‐generated inferences to established biomedical principles.

LLM‐based coordination also introduces specific risks. Biomedical LLMs can support task decomposition, tool routing, literature interpretation, and communication among agents, but they remain vulnerable to factual errors, unsupported reasoning, and hallucinated tool use. For this reason, the Full‐Body AI Agent should restrict LLMs mainly to orchestration and communication roles, while delegating quantitative analysis and mechanistic inference to validated domain‐specific tools. Schema‐constrained interfaces, inter‐hop validation, physiology‐first arbitration, and traceable reasoning graphs will be necessary to prevent unsupported intermediate claims from propagating across biological levels.

Ethical and governance considerations are equally critical. The integration of patient‐specific, multi‐modal data amplifies privacy, security, and bias concerns. Compliance with frameworks such as GDPR (General Data Protection Regulation) and HIPAA (Health Insurance Portability and Accountability Act) must be embedded by design, alongside robust encryption, anonymization, and bias‐mitigation protocols. Without careful attention to data diversity, AI‐driven recommendations risk perpetuating or exacerbating health inequities. Moreover, the framework should be positioned as an augmentative tool, with final decision‐making authority remaining firmly in the hands of medical professionals.

Looking forward, clinical translation represents the most promising but also most demanding frontier. In personalized medicine, the framework could support future modeling of individual biology at multiple scales to optimize treatment selection, dosing, and timing. In drug discovery, it could help connect molecular docking, organoid simulation, and whole‐body pharmacodynamics within a phenotype‐oriented reasoning cycle, reducing both cost and time‐to‐clinic only if validated in future studies. However, these applications will require rigorous, prospective validation to ensure that computational predictions reliably translate into therapeutic benefit.

Future work should move from conceptual design to benchmarked implementation. Key priorities include building minimal runnable prototypes for focused biological questions, testing two‐ or three‐level reasoning chains with real inputs, comparing the framework against simpler orchestration baselines, and reporting system‐level metrics such as task‐decomposition fidelity, cross‐scale alignment, arbitration success, convergence behavior, latency, cost, and failure rate. Only through such evaluations can the practical value of the seven‐layer architecture be assessed relative to simpler alternatives.

Ultimately, the Full‐Body AI‐Agent should be seen less as a fixed technological artifact and more as an evolving coordination paradigm, one capable of integrating disparate data streams across biological levels, harmonizing experimental and computational biology through task orchestration, and continuously refining models via iterative evidence‐based loops. If its technical, methodological, and ethical challenges can be addressed, it may help bridge the gap between basic biological discovery and clinically actionable precision medicine, rather than serving as a currently validated platform for clinical decision‐making.

## Funding

This work was partially supported by Center of Excellence—International Collaboration Initiative Grant (139170052), West China Hospital, Sichuan University; and National Institutes of Health [R01LM014156, R01CA241930, R01AA032723, and R01GM153822 (X.Z.)]; National Science Foundation [2217515 and 2326879 (X.Z.)]; Cancer Prevention and Research Institute of Texas [RP250043 (X.Z.)]; and Dr. & Mrs. Carl V. Vartian Professorship (X.Z.).

## Conflicts of Interest

All authors declare that they have no known competing financial interests or personal relationships that could have appeared to influence the work reported in this manuscript.

## Supporting information




**Supporting File 1**: advs75822‐sup‐0001‐TableS1‐S8.xlsx.

**Supporting File 2**: advs75822‐sup‐0002‐FigureS1‐S7.docx.

## Data Availability

The authors have nothing to report.
