# Supplementary material for: Full‐Body AI Agent: A Perspective on Multi‐Scale Collaborative AI for Systemic Biology and Precision Medicine
Source: Adv Sci (Weinh). 2026 May 26;13(36):e20562. doi: 10.1002/advs.202520562 (PMC13317560; doi:10.1002/advs.202520562)
Supplement: Supplementary file 2 — Supporting File 2: advs75822‐sup‐0002‐FigureS1‐S7.docx. [file ADVS-13-e20562-s001.docx]

- 1. **Molecule AI Agent**

Living organisms consist of a wide array of biological macromolecules, such as DNA, RNA, lipids, carbohydrates, and proteins. Each of these biomolecules plays a critical role in cellular function, and their interactions are essential for the maintenance of life. DNA encodes the genetic instructions required for development, function, and reproduction. RNA serves as a messenger, translating the genetic information encoded in DNA into proteins, which execute the majority of cellular processes. Lipids form structural components of cell membranes and participate in signaling pathways, while carbohydrates are primarily involved in energy storage and cell communication. Despite the distinct roles of these macromolecules, they are all interconnected in the regulation of cellular and organismal functions. DNA transcription into RNA leads to the production of proteins, which perform most of the cell's functional tasks. Proteins are the ultimate effectors of gene expression, and their actions are directly tied to an organism's phenotype, influencing everything from cellular metabolism to physical traits. The dynamic interactions between DNA, RNA, and proteins determine how a cell responds to its environment and how an organism develops and adapts to changes[1]. Among these biomolecules, proteins stand out because they are the direct executors of cellular function and phenotype [75]. While DNA carries the blueprint for life, it is the proteins that bring this blueprint to life by catalyzing biochemical reactions, transmitting signals, providing structural support, and regulating gene expression.

As one of the molecule AI Agents, the Protein AI-Agent is designed to understand and simulate molecular-level interactions, particularly focusing on proteins. It interprets and manipulates molecular data to predict protein structures, interactions, and biological functions. Proteins are essential biological macromolecules composed of amino acid sequences that fold into specific three-dimensional structures. These structures are critical to their diverse biological functions, which include catalyzing biochemical reactions and providing structural support to transmit signals, and defending against pathogens. The functions of proteins can be broadly categorized into several key biological domains, including protein structure and function, protein-protein interactions, post-translational modifications (PTMs), and protein design and engineering. **Figure S1** shows the predefined detachable biological tasks of the Molecule AI Agent.

The area of protein structure and function explores how amino acid sequences determine protein folding, stability, and overall function. Understanding how proteins fold into their three-dimensional structures from sequence data is crucial for uncovering their biological roles. Protein-protein interactions, which often involve the formation of protein complexes that regulate key cellular processes, are also a central focus of the Molecule AI Agent. The agent predicts these interactions and evaluates their influence on cellular functions. PTMs play a critical role in modulating protein activity, stability, and localization after translation. The Molecule AI Agent identifies potential PTM sites and simulates their effects on protein behavior and function. Additionally, the agent supports protein design and engineering, including designing proteins with specific functions, optimizing protein sequences for improved stability and efficiency, and engineering proteins for therapeutic or industrial applications.

For protein structure and function, the agent predicts folding patterns and simulates structural changes in response to mutations or environmental conditions. In protein-protein interaction analysis, it predicts how proteins interact to form complexes and assesses the implications of these interactions on cellular processes. The agent also identifies potential PTM sites and simulates the effects of these modifications on protein stability and function. In protein functional annotation, it annotates proteins with functional domains, classifies them into families, and predicts enzyme activity, binding affinity, and the effects of genetic mutations on protein function. In protein design and engineering, the agent designs proteins with specific functions, optimizes sequences for better stability and catalytic efficiency, and engineers proteins to target specific biological processes. These capabilities are crucial for understanding protein biology and have significant applications in drug discovery, disease modeling, and synthetic biology, and all of these biological tasks can be achieved by LLMs [2].

- 1. **Organelle AI Agent**

Organelle AI agents are advanced platforms designed to integrate multi-modal data and AI technologies to explore the diversity, biological functions, and dynamic behaviors of various cellular organelles. These organelles are specialized subcellular structures that perform vital functions critical to cellular health and organismal homeostasis. The most well-known organelles include the nucleus, mitochondria, endoplasmic reticulum, Golgi apparatus, lysosomes, peroxisomes, and centrosomes, among others. Each of these plays distinct roles in cellular processes, such as protein synthesis, energy production, waste management, and cell division. Within this framework, the Organelle AI Agent is specifically designed to explore the diversity, biological functions, and dynamic behaviors of Organelle in both healthy and diseased conditions.

**Figure S2** shows the predefined detachable biological tasks of the Organelle AI Agent. By providing a comprehensive description of one of the cell organelles, namely the mitochondrion, we hereby demonstrate how the Organelle AI Agent understands the functions of cell organelles and conducts biological analyses. Mitochondria are essential organelles responsible for ATP production via oxidative phosphorylation (OXPHOS), thereby fulfilling the cellular energy requirement [3]. Beyond energy production, mitochondria are central to the regulation of apoptosis, signal transduction, immune responses, and redox balance. Their unique circular mitochondrial DNA (mtDNA), characterized by a high mutation rate, has been strongly associated with a variety of diseases, including neurodegenerative disorders [4], cancers [5, 6], metabolic syndromes [7], and immune-related diseases [8]. Recent research has expanded the focus of mitochondrial studies beyond their traditional functions to include their dynamic behaviors, intercellular transfer, and molecular characteristics, revealing the multifaceted roles mitochondria play in cellular biology [9].

The Organelle AI Agent addresses several research domains within the study of mitochondria, including their morphology, distribution, lineage tracing, heterogeneity, and transfer. Within these domains, the AI Agent performs a wide range of tasks to deepen our understanding of mitochondrial functions and behaviors. It predicts the presence of mitochondrial DNA variants and assesses their potential pathogenicity, offering insights into genetic factors associated with disease. The agent also predicts mitochondrial protein structures and their interactions, contributing to the understanding of mitochondrial mechanisms at the molecular level. Additionally, the Organelle AI Agent models key mitochondrial activities, including energy metabolism, membrane potential, neuronal activity, and apoptosis pathways, simulating how mitochondria contribute to cellular processes. It also predicts core mitochondrial functions, including ATP generation efficiency, calcium ion regulation, reactive oxygen species (ROS) control, and other dynamic processes by integrating multi-omics data. Furthermore, the agent studies mitochondrial behaviors such as fusion and fission dynamics, regulation of nuclear gene expression, and inter-organelle communication, all of which are crucial for maintaining cellular health and homeostasis.

To accomplish these tasks, the Organelle AI Agent leverages cutting-edge AI tools tailored to specific aspects of mitochondrial functions. For example, tools like DeepMito [10] are used for advanced microscopy image analysis, while MitoMap [11] supports mtDNA sequencing and pathogenicity scoring. MitoTrace [12] is used to track mitochondrial variants. MitoRelID (Mitochondrial Related Interaction Descriptor) facilitates the assessment of mitochondrial drug-target interactions, enabling personalized drug discovery and treatment strategies. By integrating these tools, the Organelle AI Agent generates high-resolution annotations of mitochondrial disease characteristics, constructs dynamic models of mitochondrial transfer networks, predicts potential biomarkers for diseases, and identifies therapeutic targets. The ultimate goal of the Organelle AI Agent is to significantly enhance our understanding of mitochondrial dysfunction at molecular, cellular, and systemic levels. For instance, by evaluating mitochondrial ATP generation efficiency and energy metabolism, the agent can uncover how cancer cells adapt to hypoxic conditions. Similarly, by predicting mitochondrial transfer behaviors, it can explore strategies to prevent the propagation of damaged mitochondria or promote the transfer of healthy mitochondria, with potential implications for improving therapeutic outcomes.

- 1. **Cell AI Agent**

Cells are the fundamental building blocks of all living organisms, representing the smallest units of life capable of independently carrying out the essential processes required for an organism's survival. At the core of cellular function, the interactions among DNA, RNA, and proteins enable cells to perform a broad range of vital tasks necessary for life. DNA stores genetic information, RNA transfers this information, and proteins execute the diverse functions required to maintain cellular operations. Core cellular functions encompass energy production and metabolism, signal reception and transduction, protein synthesis and secretion, cell division and growth, DNA repair and maintenance, immune defense, transport and communication, cellular homeostasis, and mechanical movement and contraction. Advancements in high-throughput sequencing technologies have significantly enhanced our ability to examine and understand the molecular mechanisms underlying these fundamental cellular functions. These technologies enable the analysis of gene expression, protein function, metabolite profiles, and genomic integrity, providing powerful tools for studying cellular processes in both healthy and diseased conditions. The Cell AI Agent is designed to integrate multi-modal data, including genomics, proteomics, morphomics, single-cell multi-omics, and spatial omics, with deep learning algorithms to simulate and predict cellular functions, behaviors, molecular features, heterogeneities, and interactions. By incorporating clinical data, this agent bridges the gap between basic cellular research and clinical applications, facilitating disease diagnosis and treatment.

The Cell AI Agent operates across multiple core domains, beginning with the construction of intracellular metabolic networks to model biochemical pathways. It predicts cellular responses to environmental changes, such as hypoxia or toxin exposure, and links molecular dysfunctions to clinical phenotypes and disease associations. In exploring cellular behaviors, agent models processes, including cell dissemination patterns, cycle progression, differentiation trajectories, and the dynamics of apoptosis and autophagy. It also provides insights into the spatial distribution and composition of cells within tissues, contributing to tissue modeling and developmental studies. On the molecular level, the agent predicts gene expression profiles and regulatory networks, detects both genetic and epigenetic variations, and evaluates the pathogenicity of mutations. This functionality is crucial for precision medicine and the identification of drug targets. The agent also excels at analyzing cellular heterogeneity by classifying cell subtypes, predicting their functions, mapping gene expression throughout the cell life cycle, and identifying functional subtypes within populations. This is critical for understanding complex processes such as cancer evolution and immune responses. A key strength of the Cell AI Agent lies in modeling cellular interactions. It simulates cell-cell signaling, cell-matrix interactions, and molecular changes during intercellular communication. By predicting ligand-receptor interactions, the agent helps elucidate mechanisms involved in immune regulation, tissue repair, and tumor microenvironments. Clinically, the Cell AI agent contributes by identifying disease biomarkers, predicting drug responses at the cellular level, and optimizing therapeutic molecules, including antibodies and gene editors. It also supports the design of cell-based therapies such as CAR-T cells, predicts disease risks, and assesses immune evasion strategies in cancer. **Figure S3** shows the predefined detachable biological tasks assigned to the Cell AI agent.

The integration of single-cell multi-omics data is one key strength of the Cell AI Agent. By combining gene expression, chromatin accessibility, DNA methylation, and protein activity, the agent offers a comprehensive view of cellular states and functions. This multi-omics approach helps overcome challenges such as technical noise and batch effects, enabling more robust analysis and interpretation of cellular biology. Through the integration of multi-scale data and the application of advanced computational methods, the Cell AI Agent significantly enhances our understanding of cellular processes and fosters innovations in diagnostics, personalized treatment, and regenerative medicine.

To support these applications, the Cell AI Agent employs advanced computational algorithms designed for tasks such as cell clustering, cell type annotation, cell cycle modeling, and the analysis of cell-cell interactions. Clustering methods, including graph-based and density-based approaches, enable the identification of cell groups with similar gene expression profiles. Among these, the Louvain and Leiden algorithms are particularly effective for clustering in scRNA-seq analysis. Following clustering, cell type annotation is performed by assigning biological identities to each cluster based on known marker genes. Reference databases like CellMarker [13] and PanglaoDB [14] provide essential resources for both manual and automated annotation. Furthermore, tools like Monocle[15] and STREAM [16] allow researchers to reconstruct developmental trajectories and visualize gene expression patterns along pseudotime, offering valuable insights into cellular transitions and lineage relationships. To enhance these capabilities, the cell-level AI Agent further incorporates advanced AI-based tools specifically designed to overcome major challenges in single-cell analysis. For example, to address data sparsity in scRNA-seq, generative models such as Generative Adversarial Networks (GANs) [17] have been applied for imputation. To better capture dynamic processes such as the cell cycle, self-supervised learning frameworks [18] [19] have been proposed to model its circular nature and represent pseudotime along this trajectory. Similarly, novel extensions of RNA velocity, such as the lazy probability model [20], provide the ability to estimate not only the directionality but also the probability of cell state transitions. Beyond transcriptomics, transformer-based large language models (LLMs) have recently been applied to predict drug sensitivity [21], highlighting the expanding role of cell-level AI Agent in integrating diverse data modalities.

- 1. **Tissue AI Agent**

The Tissue AI Agent is an advanced computational platform designed to study tissue-level biological processes by integrating multi-omics data and spatial biology. Tissues are composed of groups of similar cells that work together to perform specific functions, often embedded within an extracellular matrix (ECM) that provides structural support and biochemical signaling necessary for tissue integrity and function. At the tissue level, the interactions between cells, the ECM, and molecular signals are crucial for maintaining homeostasis and responding to stress or disease. Understanding tissue structure and function requires the analysis of cellular interactions, molecular signaling pathways, and tissue-level dynamics. The Tissue AI Agent bridges the gap between molecular, cellular, and organ-level studies by leveraging AI-driven approaches. The Tissue AI Agent performs several key tasks, including modeling and simulation of tissue dynamics, discovery of novel biomarkers and regulatory networks, construction of comprehensive tissue knowledge bases, and prediction of patient-specific tissue responses to treatments. In tissue dynamics, the agent integrates spatial omics data and agent-based modeling to simulate tissue environments. This enables a deeper understanding of how tissues develop, maintain homeostasis, and respond to perturbations such as injury or disease. For example, the agent can model tissue repair following injury or track the progression of diseases like fibrosis and cancer.

Through these simulations, the Tissue AI Agent predicts emergent properties such as tissue differentiation, regenerative potential, and disease-related changes in tissue architecture. To advance tissue biology research, the agent employs self-supervised learning techniques to uncover tissue-specific regulatory networks, rare cell populations, and novel biomarkers. This autonomous discovery capability enables deeper insights into disease mechanisms and supports the development of diagnostic strategies. The Tissue AI Agent integrates multi-omics data-such as single-cell transcriptomics, proteomics, and epigenomics, with spatial mapping to construct a comprehensive tissue knowledge base. By using AI-driven knowledge graphs, it uncovers functional tissue interactions and sheds light on both healthy and diseased states. In the context of personalized medicine, the Tissue AI Agent predicts patient-specific responses to treatments by analyzing tissue heterogeneity. It can detect early disease signatures, such as those present in cancer microenvironments or neurodegenerative disorders, thereby facilitating the development of precision therapies. Furthermore, the agent contributes to biofabrication and synthetic tissue engineering by simulating stem cell differentiation and optimizing conditions for tissue regeneration. **Figure S4** shows the Tissue AI-Agent predefined detachable biological tasks performed by the Tissue AI Agent.

The Tissue AI-Agent employs several advanced AI tools and algorithms to achieve these tasks. Including: (1) Deep learning models, such as convolutional neural networks (CNNs) and Vision Transformers (ViTs), are used to analyze histopathological features and spatial omics data. SEQUOIA [22] and RNAPath [23] are transformer-based models that leverage whole slide histology images to respectively predict cancer transcriptomic profiles and spatially localize RNA expression, enabling cost-effective genomic analysis and revealing the spatial interplay between tissue morphology and gene expression. (2) Graph neural networks (GNNs) are utilized to model cell-cell interactions and tissue architecture, while reinforcement learning (RL) simulates tissue responses to external perturbations. STCase [24] and Ceograph [25] are graph neural network-based models that analyze spatial transcriptomics and pathology images, respectively, to uncover niche-specific cell-cell communication and spatial cell organization, enabling fine-grained mapping of microenvironmental interactions and accurate prediction of clinically relevant tissue features. And, RL-GenRisk [26] uses reinforcement learning to simulate tissue responses to pathological perturbations, identifying ccRCC (clear cell renal cell carcinoma) risk genes through a graph-based Markov decision process. (3) SSL techniques allow the agent to discover hidden spatial patterns within tissue samples without requiring labeled data, enabling deeper insights into tissue organization and behavior. BIDCell [27] and Kasumi [28] are self-supervised learning models that uncover spatial patterns in tissue without labeled data, BIDCell links gene expression to cell morphology for accurate segmentation, while Kasumi identifies persistent spatial neighborhoods associated with disease outcomes. By integrating these computational techniques, the Tissue AI-Agent is capable of predicting how tissues evolve in response to physiological and pathological stimuli. This ability to model tissue dynamics provides new opportunities for understanding disease progression, tissue regeneration, and therapeutic interventions, and the development of targeted therapeutic interventions.

- 1. **Organ AI Agent**

The Organ AI Agent is designed to model and analyze the biological functions of individual organs by integrating multi-omics data, clinical information, and imaging. This integration provides comprehensive insights into organ-specific diseases, functions, and responses to stimuli. The agent constructs detailed anatomical and functional models of organs, simulates their behavior in various conditions, and predicts responses to environmental changes or treatments. The Organ AI Agent focuses on several core tasks, including modeling organ function, analyzing organ pathophysiology, identifying molecular features specific to the organ, examining heterogeneity within the organ, and studying tissue interactions. Each task is based on data from multiple sources, such as genomics, transcriptomics, proteomics, physiological measurements, and imaging, which are collectively integrated to generate a holistic organ profile. **Figure S5** shows the predefined detachable biological tasks designed for the Organ AI Agent.

Organ function modeling involves simulating and predicting an organ's functional status under both healthy and diseased conditions. This includes evaluating the impact of environmental factors on organ function, such as nutrient availability, metabolic states, or disease progression. By combining anatomical imaging (e.g., MRI or CT) with molecular data, the Organ AI Agent can simulate dynamic changes in organ function over time, including responses to injury, disease, or therapeutic interventions. In organ pathophysiology analysis, the agent examines the dynamic alterations that occur in response to specific diseases. It predicts the underlying causes of organ dysfunction, providing insights into disease mechanisms and helping to identify potential therapeutic targets. For example, the agent may simulate the progression of fibrosis following chronic injury or model tumorigenesis driven by specific genetic mutations. Additionally, the Organ AI Agent also identifies molecular features associated with normal organ function and pathological states, such as particular marker genes or molecular pathways. By analyzing transcriptomics and proteomics data, the Organ AI agent can identify molecular characteristics linked to disease progression and predict how these features evolve across different disease states. Another key function of the Organ AI Agent is examining organ heterogeneity. It predicts spatial and functional variability within the organ, identifying regions with distinct molecular and cellular profiles. This analysis can be used to study tissue-specific changes, such as how different cell types, metabolic activities, or molecular distributions contribute to organ function and dysfunction. Additionally, the agent models tissue interactions within the organ. It simulates the flow of signals and materials between different tissue regions, such as the interaction of immune cells with tissues during inflammation or the influence of signaling pathways during tissue repair. By integrating spatial omics and imaging data, the Organ AI Agent provides a detailed view of how various regions of the organ function coordinate functionally and how they respond to external stimuli. Finally, the Organ AI Agent supports drug response and toxicity analysis by predicting the impacts of treatments on specific organs. It can optimize organ engineering and regenerative medicine strategies, such as organ transplantation, by simulating the adaptation process of transplanted organs in the host and predicting the outcomes of various therapeutic approaches.

The Organ AI Agent also leverages several advanced technologies and methodologies to model and analyze the dynamic interactions between organs. Organ-on-a-chip technology employs microfluidic chips to simulate in vitro organ environments and inter-organ interactions. It is ideal for dynamic physiological studies, complex disease modeling, and drug evaluation.

Organoid culture involves the generation of miniature, three-dimensional organ structures from primary tissues or stem cells under controlled conditions[29]. These models offer valuable insights into organ development, disease processes, and personalized medicine. Finite element analysis (FEA) is used to simulate and analyze the biomechanical properties of tissues, such as muscle behavior, under different conditions [30]. It can facilitate the understanding of how mechanical factors influence organ function. Virtual human modeling and simulation create detailed three-dimensional models of the human body [31], allowing for the simulation of organ movement and interactions. This is particularly useful for medical device development, surgical planning, and the design of individualized treatments. By integrating these diverse methods, the Organ AI-Agent delivers a comprehensive understanding of organ function, interactions, and responses to external factors.

Some studies have integrated imaging and genetic analysis to investigate organ function and its association with diseases, and the Organ AI-Agent will incorporate these capabilities [32, 33]. It begins with MRI and CT imaging to identify functional regions of interest (ROIs) within the organ, providing high-resolution anatomical and functional data. Once these ROIs are defined, the agent uses genome-wide association studies (GWAS) to link genetic variants to specific features within these regions, thereby identifying genetic loci that influence organ function or pathology. To infer causal relationships, Mendelian randomization (MR) is employed, using genetic variants as instrumental variables to determine if genetic factors directly affect organ function, independent of confounding variables. By integrating imaging with GWAS and Mendelian randomization, the agent constructs a comprehensive model of organ behavior, linking structural features with genetic pathways to identify potential therapeutic targets for precision medicine.

- 1. **Organ System AI Agent**

The Organ System AI Agent is an advanced platform designed to model and analyze inter-organ communication, homeostasis, and responses to perturbations across multiple organs. By integrating a vast array of biological, physiological, environmental, and molecular data through sophisticated AI models, the agent provides a comprehensive understanding of organ-to-organ interaction within the human body. Organ systems are fundamental for maintaining physiological functions, and their coordinated communication, both local and distant, is critical for adapting to diseases and ensuring overall homeostasis. Recent studies highlight the interconnection between organ systems in conditions such as cardiovascular, immune, and neurodegenerative diseases, revealing how dysfunction in one organ can influence the function of others. A notable example is the gut-brain axis, where gut-derived factors like α-Syn have been implicated in the early diagnosis of Parkinson's disease [34]. By capturing these complex inter-organ relationships, the Organ System AI Agent enables disease prediction, biomarker discovery, and optimization of therapeutic strategies.

The Organ System AI Agent integrates multiple datasets, including multi-omics (genomics, metabolomics, metagenomics), physiological data (e.g., ECG, blood glucose, sleep rhythm), anatomical data (e.g., liver thickness, left ventricular volume), whole-body imaging data (CT, MRI, ultrasonography), and environmental factors (e.g., smoking, drug use) to carry out a wide range of downstream biological tasks. Image data provides detailed anatomical structures, while non-image data offers insights into organ functions and overall health conditions. The core tasks of the Organ System AI-Agent include analyzing the coordination of multi-organ functions, modeling dysfunction in systemic diseases across organs, simulating multi-organ pathological cascades, and predicting drug responses. The agent also simulates cross-organ compensation and repair processes following injury, constructs multi-organ metabolic networks, and infers biomechanical interactions between organs to predict functional and phenotypic changes. **Figure S6** shows the predefined detachable biological tasks designed for the Organ System AI Agent.

To achieve these tasks, the Organ System AI-Agent relies on advanced AI tools such as nnU-Net for biomedical image segmentation [35], TMO-Net for capturing self-modal and cross-modal features [36], and BioBERT [37] for biomedical text mining. Additionally, Mendelian randomization is employed to identify causal relationships between organs based on genomic variants, enabling the investigation of how genetic factors influence inter-organ interactions and the development of systemic diseases. By integrating this approach with other analytical tools, the Organ System AI Agent captures the anatomical, molecular, and environmental features of organs under various biological states.

- 1. **Body system AI Agent**

The Body System AI Agent is a biological-level agent responsible for reasoning and making predictions at the organismal physiological scale. As illustrated in **Figure S7**, the Body System AI-Agent encompasses several core functions that operate both within and across organ systems, including: (1) Intra-System Functional Coordination: The Body System AI-Agent models the dynamic interplay between organs within a single physiological system. For instance, it analyzes how the heart, lungs, and kidneys coordinate to regulate circulatory and respiratory functions. By integrating imaging data (MRI, CT), electronic medical records (EMR), and multi-omics datasets (genomics, metabolomics), the agent simulates how perturbations in one organ influence others, providing insights into system-level homeostasis and organ resilience. (2) Inter-System Functional Coupling: Beyond individual systems, the agent captures crosstalk between different organ systems, such as the nervous, immune, and endocrine systems. It simulates how neural signaling affects musculoskeletal function or how hormonal cascades, from the hypothalamus to the adrenal glands, regulate physiological responses. This modeling enables discovery of regulatory axes and feedback loops that underlie whole-body function. (3) Systemic Disease Propagation: The Body System AI-Agent can predict how diseases spread across organ systems. For example, it models how chronic inflammation originating in the gut may trigger immune responses or dysfunctions in distant organs, contributing to systemic disorders like autoimmune diseases or multi-organ failure. These simulations help uncover disease pathways and inform early intervention strategies. (4) Cross-Organ Disease Dynamics: The agent simulates disease progression across organ systems, such as how cardiovascular disease may impair renal function or how diabetes can affect neural and ocular tissues. By modeling disease trajectories and interaction networks, the agent aids in identifying critical nodes for therapeutic targeting and in understanding systemic comorbidities. (5) System-Level Metabolic Dynamics: A key strength of the Body System AI-Agent lies in modeling metabolic flow between and within organ systems. It simulates how disruptions in lipid or glucose metabolism affect organs such as the liver, pancreas, and cardiovascular system. These insights are vital for studying metabolic syndromes and their systemic consequences, including obesity, type 2 diabetes, and fatty liver disease. (6) Biomechanical and Structural Interactions: The agent integrates mechanical forces and structural biology to simulate how physical stressors affect organ behavior. For instance, it can predict the heart's adaptation to pressure overload or how skeletal muscle responds to repetitive motion. This functionality supports modeling of injury, aging, and adaptive responses under different physical conditions. (7) Personalized Therapy Optimization: The Body System AI-Agent plays a central role in precision medicine by simulating individualized responses to drugs and interventions across multiple organ systems. By leveraging EMR, clinical trial data, and omics profiles, it predicts therapeutic efficacy and toxicity, optimizes combination treatments, and tailors drug regimens to maximize systemic benefit while minimizing adverse effects.

In addition, EMR data plays a crucial role in understanding human systems biology, as it captures the macroscopic manifestations of dynamic changes occurring across multiple biological levels. While numerous studies have been conducted to analyze the physiological changes of different organs or systems based on EMR data, these studies face a significant limitation: they cannot simultaneously capture all organ-level data from the same individual. This constraint hinders holistic insights into the body's systemic complexity. To address this, we propose incorporating LLMs into the Body System AI-Agent framework. By mining EMR data, LLMs can identify physiological signals associated with specific organs, shared across multiple organs, or linked to comorbid disease states. This capability enables the construction of a high-dimensional, integrated network that maps physiological signals to organs and organ systems. Through such integration, the Body System AI-Agent can reveal the interdependencies among biological systems, offering deeper insights into human health and disease and enabling more precise, system-aware therapeutic strategies.


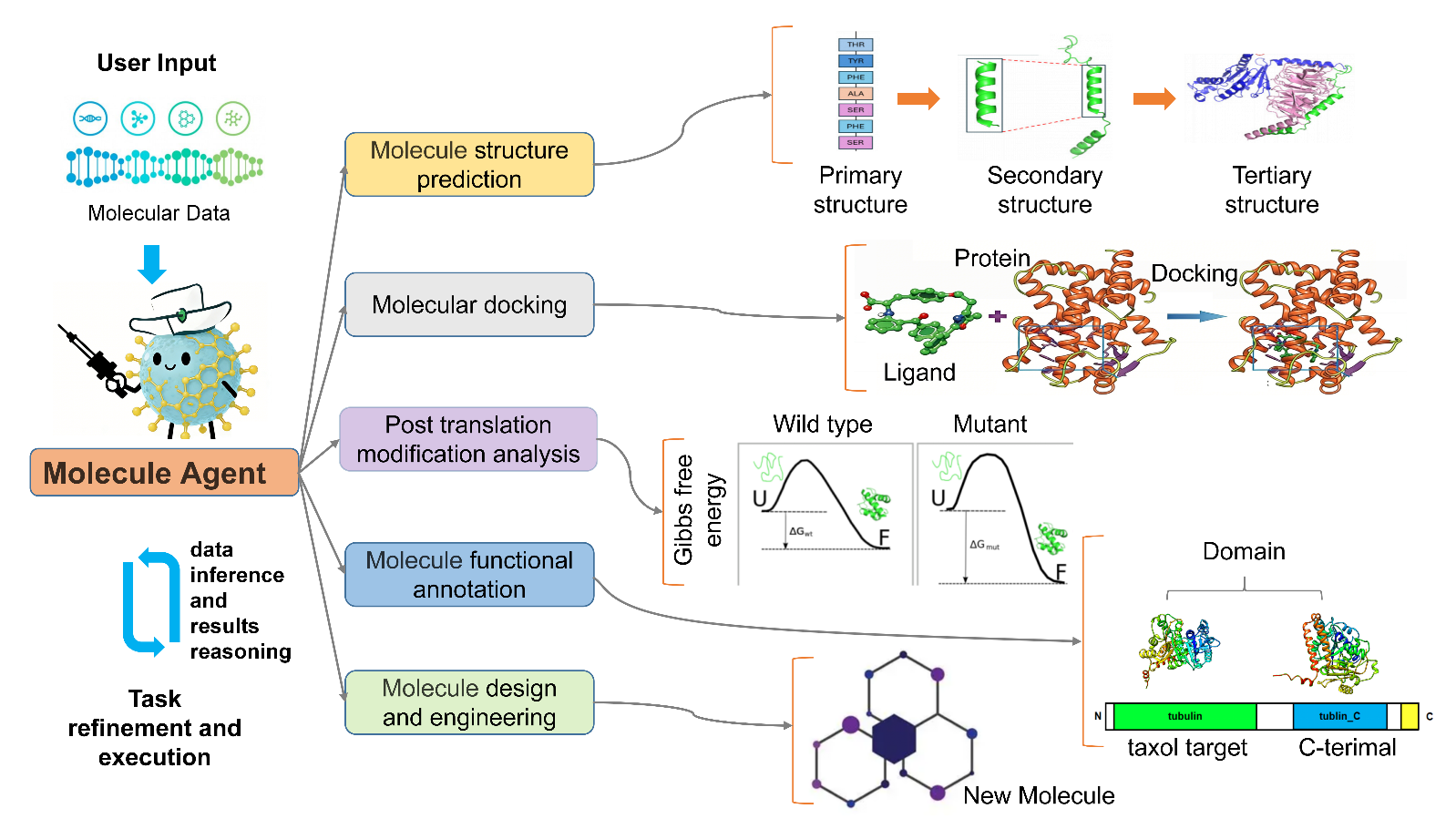


**Figure S1.** **Predefined detachable biological tasks of the Molecule AI Agent.** This figure illustrates the core capabilities of the Molecule AI Agent, which accepts molecular data as input and executes a suite of specialized, detachable tasks to support multi-scale biological reasoning. These tasks include: (1) molecule structure prediction, which infers primary, secondary, and tertiary protein structures from sequence data; (2) molecular docking, which models interactions between ligands and target proteins; (3) post-translation modification analysis, which compares Gibbs free energy changes between wild-type and mutant proteins to assess functional impacts; (4) molecule functional annotation, which identifies structural domains and functional regions (e.g., taxol target domains in tubulin); and (5) molecule design and engineering, which generates novel molecular structures. The agent iteratively refines tasks based on data inference and result reasoning, enabling targeted molecular-level analysis within the Full-Body AI-Agent framework.


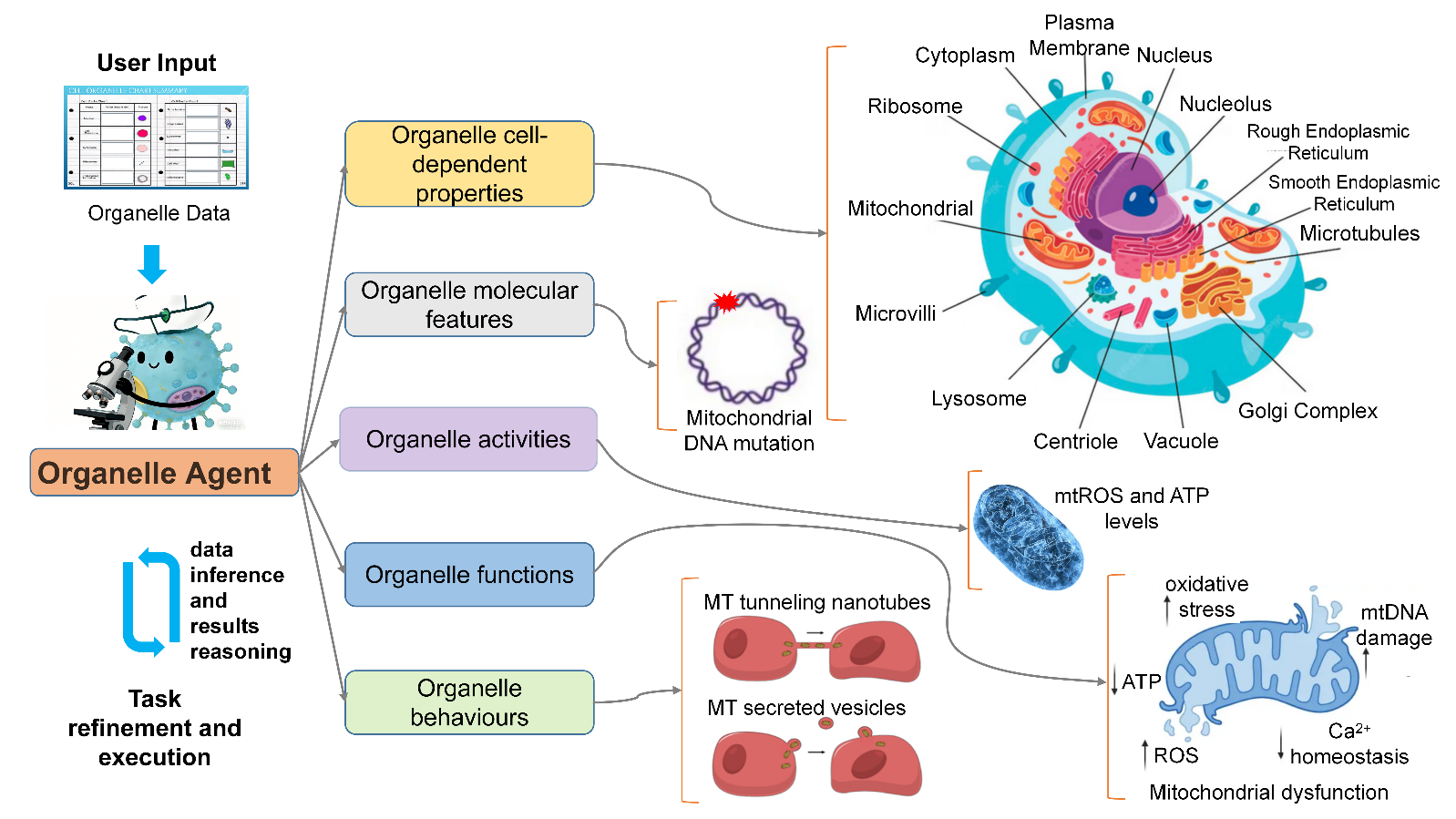


**Figure S2. Predefined detachable biological tasks of the Organelle AI-Agent.** This figure outlines the core functional repertoire of the Organelle AI Agent, which processes organelle-specific data as input and executes a series of detachable, modular biological tasks, exemplified here using mitochondria, a well-characterized organelle with established links to major human diseases. The agent’s key tasks include: (1) characterizing organelle cell-dependent properties, illustrated by the subcellular localization and structural components of mitochondria within the eukaryotic cell; (2) identifying organelle molecular features, such as mitochondrial DNA (mtDNA) mutations; (3) quantifying organelle activities, represented by the levels of mitochondrial reactive oxygen species (mtROS) and ATP; (4) annotating organelle functions, including the mechanistic consequences of mitochondrial dysfunction (e.g., oxidative stress, mtDNA damage, disrupted Ca²⁺ homeostasis); and (5) analyzing organelle behaviours, such as intercellular communication via mitochondrial tunneling nanotubes and secreted vesicles. Guided by iterative data inference, result reasoning, and task refinement, the Organelle AI Agent enables systematic analysis of organelle biology, with mitochondria serving as a prototype to illustrate its generalizable capacity to interpret the structure and function of any cell organelle within the Full-Body AI-Agent framework.


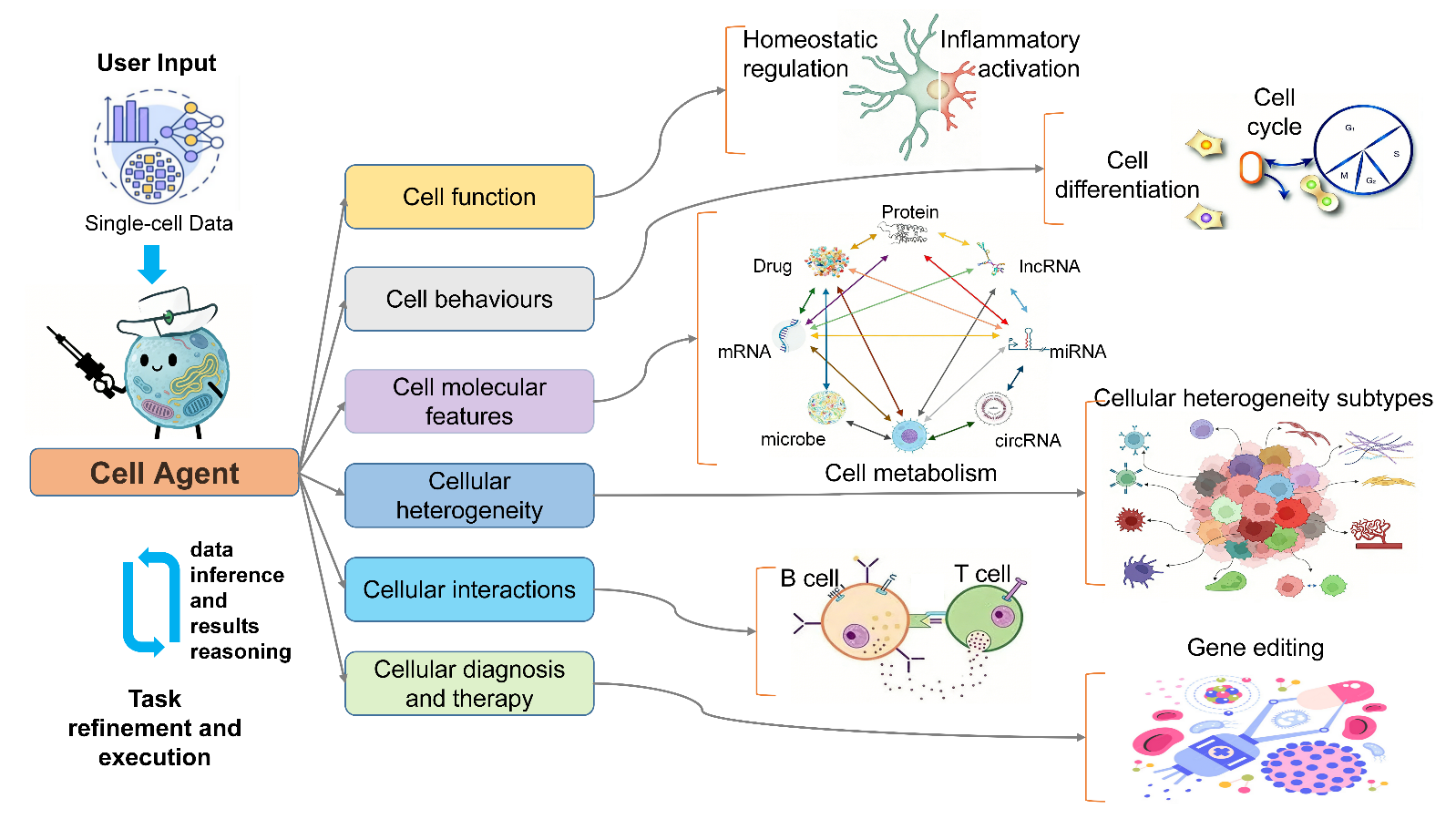


**Figure S3. The predefined detachable biological tasks assigned to the Cell AI Agent.** This figure depicts the modular task set of the Cell AI Agent, which processes cell-specific multi-modal data and executes level-specific analyses within the Full-Body AI-Agent framework. Its core functions include: (1) cell type identification and subtyping via transcriptomic and spatial data; (2) cell state and function analysis through pathway enrichment and functional annotation; (3) inference of cell–cell communication networks; (4) mapping of cellular spatial organization in tissues; and (5) tracking of cellular dynamics and plasticity. Through iterative data inference and task refinement, the agent translates raw data into multi-scale insights for systemic biological modeling.


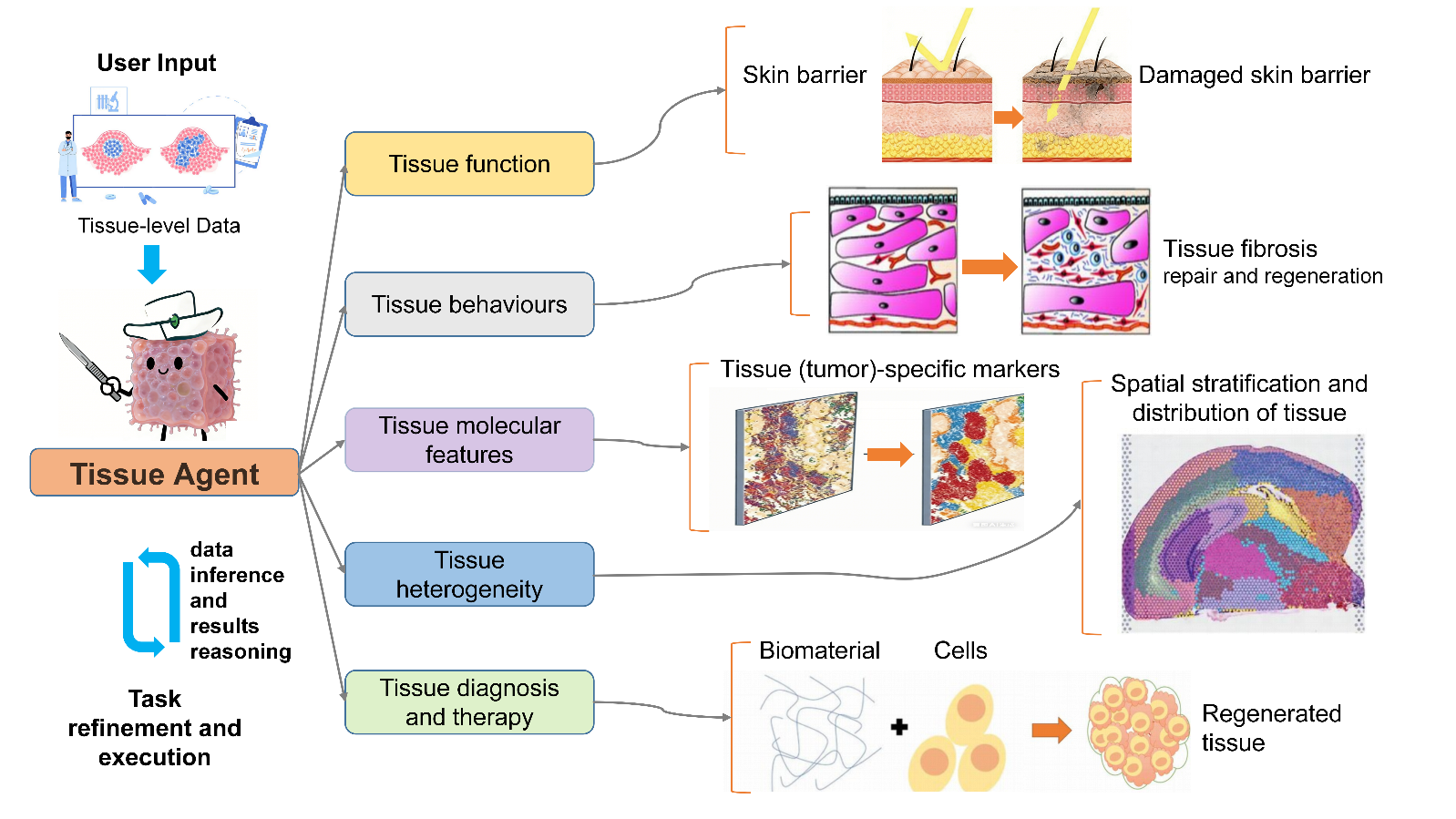


**Figure S4. The predefined detachable biological tasks for the Tissue AI Agent.** This figure outlines the modular analytical tasks of the Tissue AI Agent, which processes single-cell data to address core tissue-level biological questions, with key application domains illustrated as examples. Core tasks include characterizing cell function (e.g., homeostatic regulation, inflammatory activation), cell behaviours (e.g., differentiation, cell cycle), cell molecular features (e.g., metabolic and regulatory networks), cellular heterogeneity (e.g., subtype classification), cellular interactions (e.g., immune cell crosstalk), and cellular diagnosis and therapy (e.g., gene editing). Operating via iterative data inference, result reasoning and task refinement within the Full-Body AI-Agent framework, the agent translates single-cell inputs into tissue-scale biological insights.


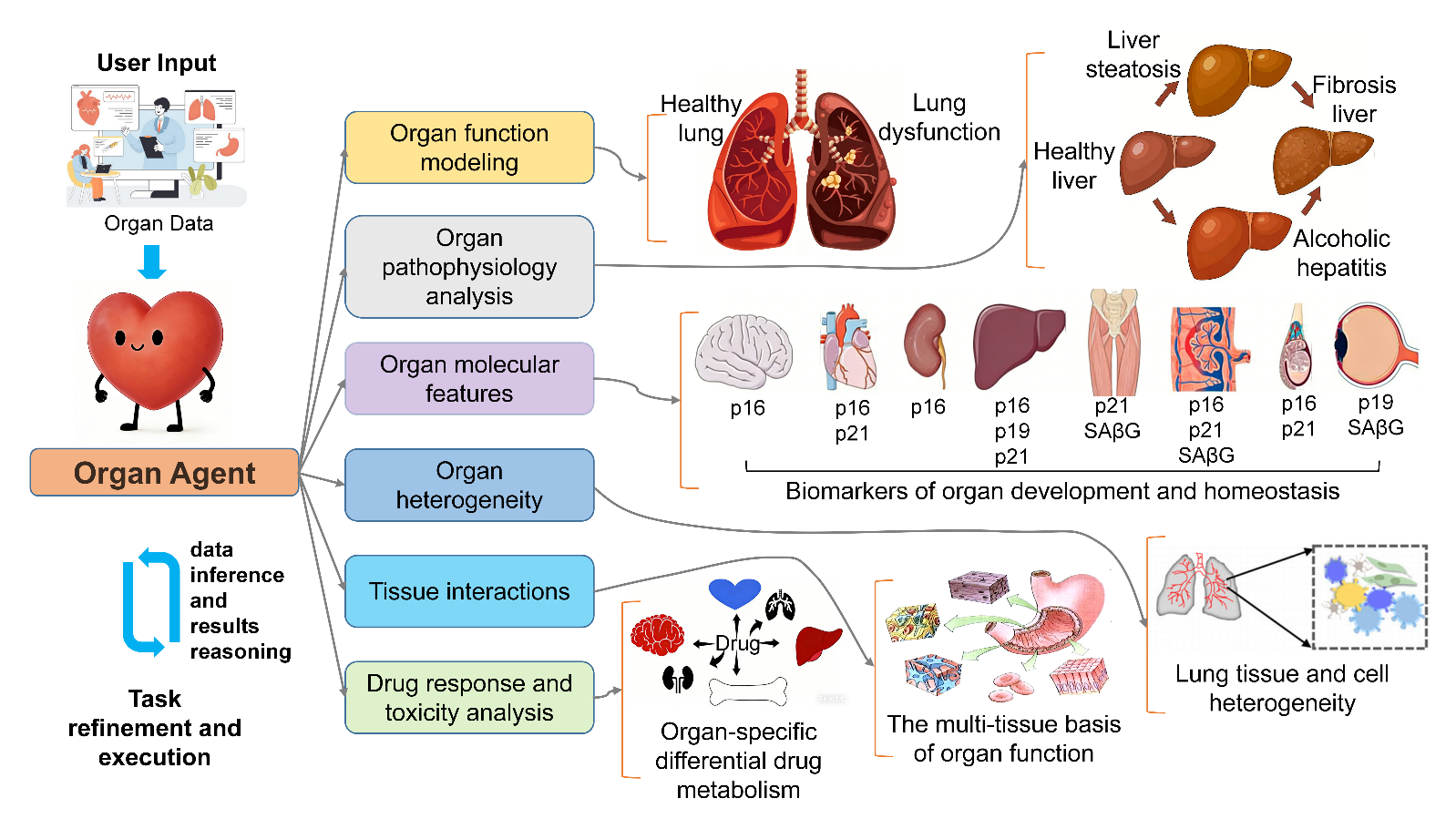


**Figure S5.** **The predefined detachable biological tasks performed by the Organ AI Agent.** This figure illustrates the modular task repertoire of the Organ AI Agent, which processes organ-specific clinical and molecular data as input and executes a suite of detachable, level-specific analyses within the Full-Body AI-Agent framework. Key tasks include: (1) organ function modeling, exemplified by comparing healthy and dysfunctional lung and liver states (e.g., steatosis, fibrosis, alcoholic hepatitis); (2) organ pathophysiology analysis, modeling disease progression across organs; (3) organ molecular features, identifying biomarkers (e.g., p16, p21, SAβG) of organ development and homeostasis across multiple organs; (4) organ heterogeneity, mapping tissue and cell-level diversity (e.g., lung tissue heterogeneity); (5) tissue interactions, revealing the multi-tissue basis of organ function; and (6) drug response and toxicity analysis, modeling organ-specific differential drug metabolism. Through iterative data inference, result reasoning, and task refinement, the Organ AI Agent translates organ-level data into actionable insights for systemic disease modeling and precision medicine.


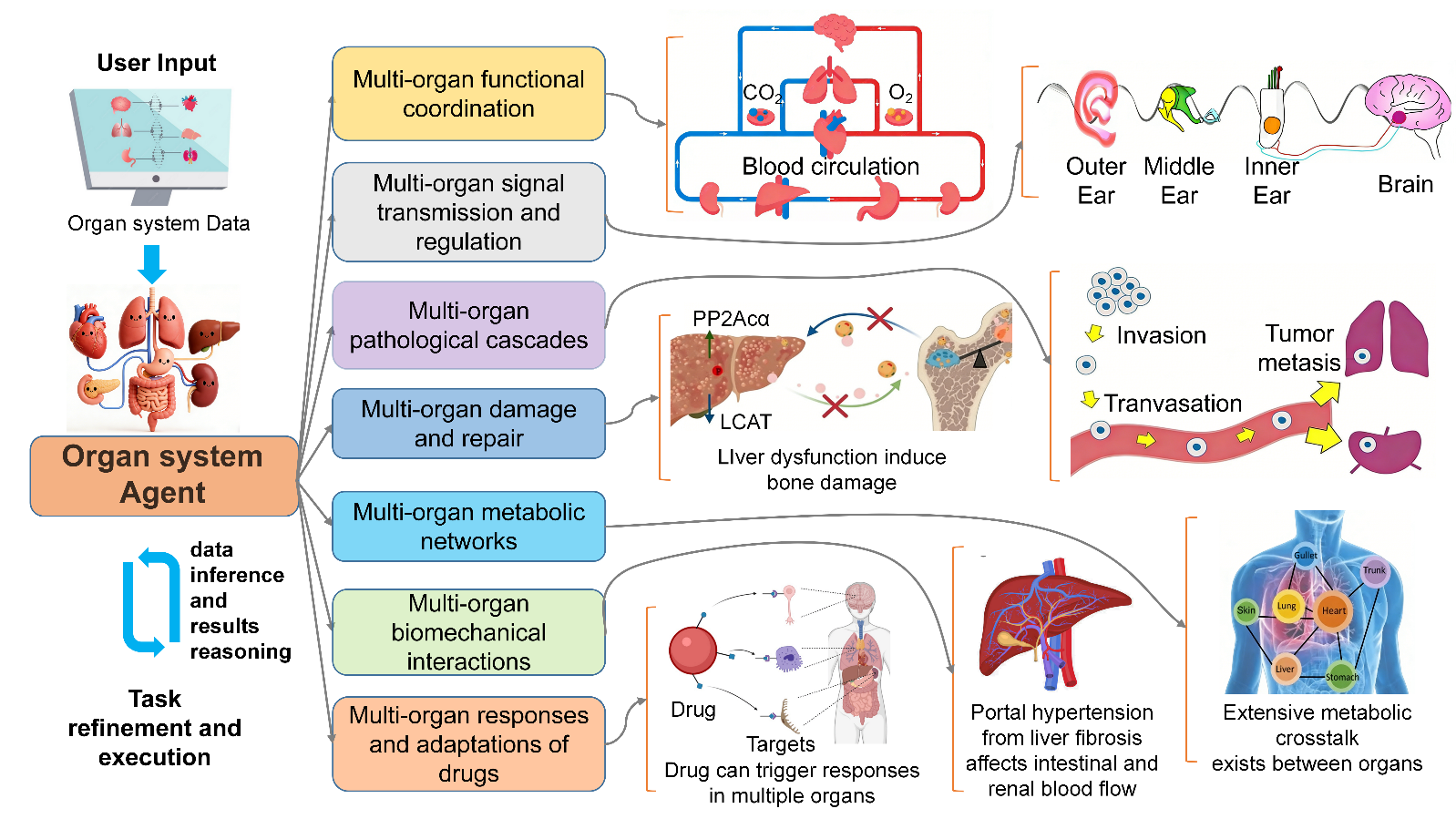


**Figure S6.** **Predefined detachable biological tasks for the Organ System AI Agent.** This figure illustrates the modular task repertoire of the Organ System AI Agent, which processes multi-organ system data as input and executes a suite of detachable, level-specific analyses within the Full-Body AI-Agent framework. Key tasks include: (1) multi-organ functional coordination, modeling systemic processes like blood circulation and sensory signal transmission (e.g., auditory pathways from the ear to the brain); (2) multi-organ signal transmission and regulation, integrating inter-organ communication networks; (3) multi-organ pathological cascades, modeling disease progression such as liver dysfunction-induced bone damage and tumor metastasis; (4) multi-organ damage and repair, analyzing systemic injury and regenerative responses; (5) multi-organ metabolic networks, mapping extensive metabolic crosstalk between organs (e.g., portal hypertension affecting intestinal and renal blood flow); (6) multi-organ biomechanical interactions, modeling physical and functional interdependencies; and (7) multi-organ responses and adaptations of drugs, predicting systemic drug effects across multiple organs. Through iterative data inference, result reasoning, and task refinement, the Organ System AI Agent translates multi-organ data into systemic insights for disease modeling and therapeutic development.


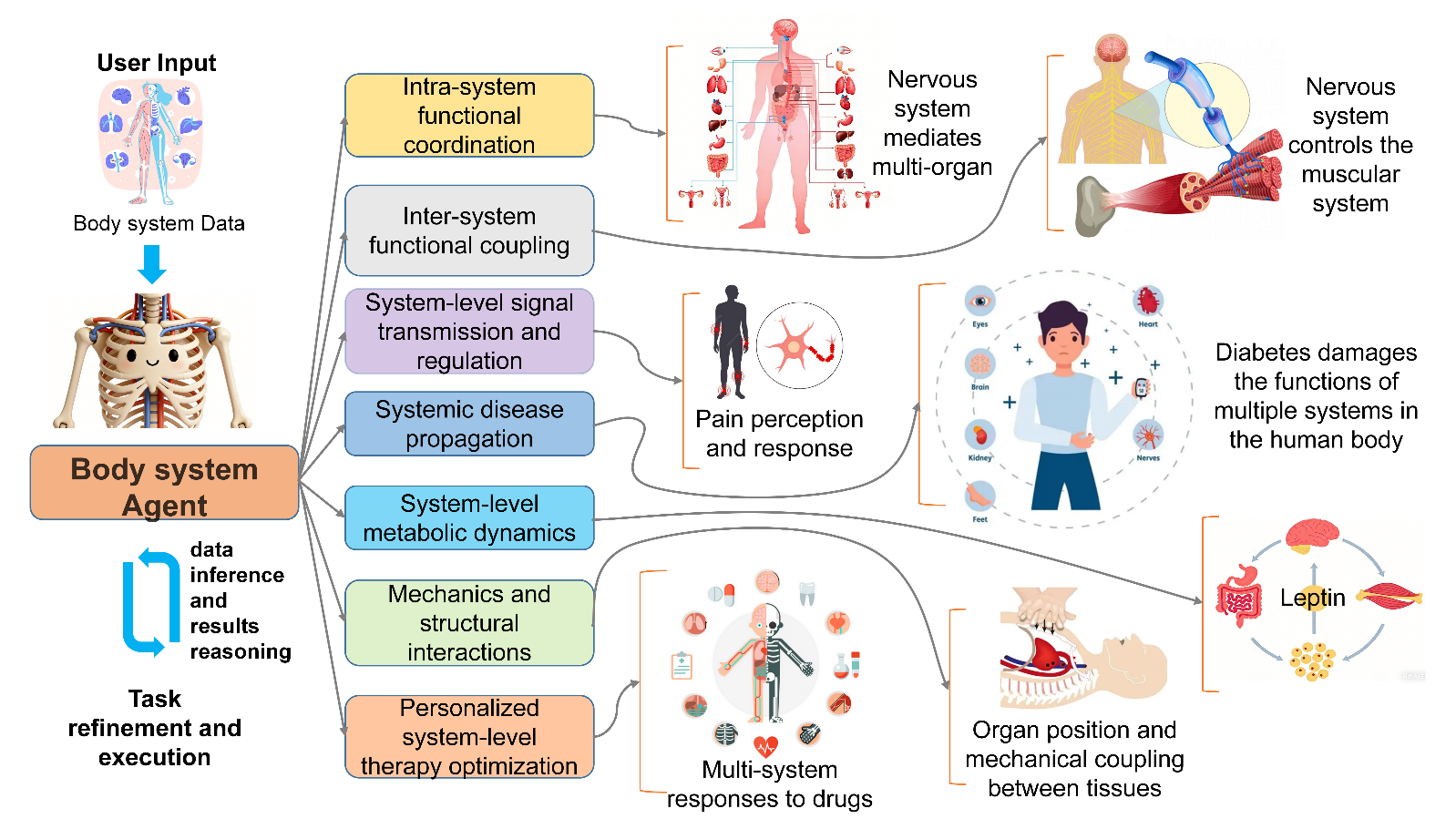


**Figure S7. Predefined detachable biological tasks for the Body System AI Agent.** This figure illustrates the modular task repertoire of the Body System AI Agent, which processes whole-body system data as input and executes a suite of detachable, level-specific analyses within the Full-Body AI-Agent framework. Key tasks include: (1) intra-system functional coordination, modeling how the nervous system mediates multi-organ function and controls the muscular system; (2) inter-system functional coupling, integrating cross-system interactions; (3) system-level signal transmission and regulation, exemplified by pain perception and response pathways; (4) systemic disease propagation, modeling multi-system dysfunction such as diabetes-related damage across neural, cardiac, renal, and metabolic pathways; (5) system-level metabolic dynamics, mapping inter-organ signaling (e.g., leptin-mediated gut-brain-adipose crosstalk); (6) mechanics and structural interactions, analyzing organ position and mechanical coupling between tissues; and (7) personalized system-level therapy optimization, predicting multi-system responses to drugs and refining therapeutic strategies. Through iterative data inference, result reasoning, and task refinement, the Body System AI Agent translates whole-body data into systemic insights for precision medicine and disease modeling.

**Reference**

[1] Y. Yan and T. Huang, "The Interactome of Protein, DNA, and RNA," (in eng), *Methods Mol Biol,* vol. 2695, pp. 89-110, 2023, doi: 10.1007/978-1-0716-3346-5_6.

[2] J. Liu *et al.*, "Advancing bioinformatics with large language models: components, applications and perspectives," (in eng), *ArXiv,* Jan 31 2025.

[3] L. E. Formosa and M. T. Ryan, "Mitochondrial OXPHOS complex assembly lines," *Nature Cell Biology,* vol. 20, no. 5, pp. 511-513, 2018/05/01 2018, doi: 10.1038/s41556-018-0098-z.

[4] M. M. Klemmensen, S. H. Borrowman, C. Pearce, B. Pyles, and B. Chandra, "Mitochondrial dysfunction in neurodegenerative disorders," (in eng), *Neurotherapeutics,* vol. 21, no. 1, p. e00292, Jan 2024, doi: 10.1016/j.neurot.2023.10.002.

[5] P. K. Kopinski, L. N. Singh, S. Zhang, M. T. Lott, and D. C. Wallace, "Mitochondrial DNA variation and cancer," *Nature Reviews Cancer,* vol. 21, no. 7, pp. 431-445, 2021/07/01 2021, doi: 10.1038/s41568-021-00358-w.

[6] "Mitochondrial DNA mutation enhances sensitivity to immunotherapy in melanoma," *Nature Cancer,* vol. 5, no. 4, pp. 544-545, 2024/04/01 2024, doi: 10.1038/s43018-023-00722-9.

[7] J. A. Amorim, G. Coppotelli, A. P. Rolo, C. M. Palmeira, J. M. Ross, and D. A. Sinclair, "Mitochondrial and metabolic dysfunction in ageing and age-related diseases," *Nature Reviews Endocrinology,* vol. 18, no. 4, pp. 243-258, 2022/04/01 2022, doi: 10.1038/s41574-021-00626-7.

[8] M.-M. Hu and H.-B. Shu, "Mitochondrial DNA-triggered innate immune response: mechanisms and diseases," *Cellular & Molecular Immunology,* vol. 20, no. 12, pp. 1403-1412, 2023/12/01 2023, doi: 10.1038/s41423-023-01086-x.

[9] A. S. Monzel, J. A. Enríquez, and M. Picard, "Multifaceted mitochondria: moving mitochondrial science beyond function and dysfunction," *Nature Metabolism,* vol. 5, no. 4, pp. 546-562, 2023/04/01 2023, doi: 10.1038/s42255-023-00783-1.

[10] C. Savojardo, N. Bruciaferri, G. Tartari, P. L. Martelli, and R. Casadio, "DeepMito: accurate prediction of protein sub-mitochondrial localization using convolutional neural networks," (in eng), *Bioinformatics,* vol. 36, no. 1, pp. 56-64, Jan 1 2020, doi: 10.1093/bioinformatics/btz512.

[11] A. M. Kogelnik, M. T. Lott, M. D. Brown, S. B. Navathe, and D. C. Wallace, "MITOMAP: a human mitochondrial genome database," (in eng), *Nucleic Acids Res,* vol. 24, no. 1, pp. 177-9, Jan 1 1996, doi: 10.1093/nar/24.1.177.

[12] M. Wang, W. Deng, D. C. Samuels, Z. Zhao, and L. M. Simon, "MitoTrace: A Computational Framework for Analyzing Mitochondrial Variation in Single-Cell RNA Sequencing Data," (in eng), *Genes (Basel),* vol. 14, no. 6, Jun 4 2023, doi: 10.3390/genes14061222.

[13] X. Zhang *et al.*, "CellMarker: a manually curated resource of cell markers in human and mouse," (in eng), *Nucleic Acids Res,* vol. 47, no. D1, pp. D721-d728, Jan 8 2019, doi: 10.1093/nar/gky900.

[14] O. Franzén, L. M. Gan, and J. L. M. Björkegren, "PanglaoDB: a web server for exploration of mouse and human single-cell RNA sequencing data," (in eng), *Database (Oxford),* vol. 2019, Jan 1 2019, doi: 10.1093/database/baz046.

[15] X. Qiu *et al.*, "Reversed graph embedding resolves complex single-cell trajectories," *Nat Methods,* vol. 14, no. 10, pp. 979-982, 2017/10/01 2017, doi: 10.1038/nmeth.4402.

[16] H. Chen *et al.*, "Single-cell trajectories reconstruction, exploration and mapping of omics data with STREAM," (in eng), *Nat Commun,* vol. 10, no. 1, p. 1903, Apr 23 2019, doi: 10.1038/s41467-019-09670-4.

[17] Y. Xu, Z. Zhang, L. You, J. Liu, Z. Fan, and X. Zhou, "scIGANs: single-cell RNA-seq imputation using generative adversarial networks," *Nucleic Acids Research,* vol. 48, no. 15, pp. e85-e85, 2020, doi: 10.1093/nar/gkaa506.

[18] J. Liu, M. Yang, W. Zhao, and X. Zhou, "CCPE: cell cycle pseudotime estimation for single cell RNA-seq data," *Nucleic Acids Research,* vol. 50, no. 2, pp. 704-716, 2021, doi: 10.1093/nar/gkab1236.

[19] J. Liu, J. Ma, J. Wen, and X. Zhou, "A Cell Cycle-Aware Network for Data Integration and Label Transferring of Single-Cell RNA-Seq and ATAC-Seq," *Advanced Science,* vol. n/a, no. n/a, p. 2401815, 2024, doi: <https://doi.org/10.1002/advs.202401815>.

[20] S. Mao, J. Liu, W. Zhao, and X. Zhou, "LVPT: Lazy Velocity Pseudotime Inference Method," *Biomolecules,* vol. 13, no. 8, Aug 12 2023, doi: 10.3390/biom13081242.

[21] X. Liu *et al.*, "DrugFormer: Graph-Enhanced Language Model to Predict Drug Sensitivity," *Adv Sci (Weinh),* vol. 11, no. 40, p. e2405861, Oct 2024, doi: 10.1002/advs.202405861.

[22] M. Pizurica *et al.*, "Digital profiling of gene expression from histology images with linearized attention," *Nature Communications,* vol. 15, no. 1, p. 9886, 2024/11/14 2024, doi: 10.1038/s41467-024-54182-5.

[23] F. Cisternino, S. Ometto, S. Chatterjee, E. Giacopuzzi, A. P. Levine, and C. A. Glastonbury, "Self-supervised learning for characterising histomorphological diversity and spatial RNA expression prediction across 23 human tissue types," *Nature Communications,* vol. 15, no. 1, p. 5906, 2024/07/13 2024, doi: 10.1038/s41467-024-50317-w.

[24] J. Qi, Z. Luo, C.-Y. Li, J. Wang, and W. Ding, "Interpretable niche-based cell‒cell communication inference using multi-view graph neural networks," *Nature Computational Science,* 2025/05/27 2025, doi: 10.1038/s43588-025-00809-6.

[25] S. Wang *et al.*, "Deep learning of cell spatial organizations identifies clinically relevant insights in tissue images," *Nature Communications,* vol. 14, no. 1, p. 7872, 2023/12/11 2023, doi: 10.1038/s41467-023-43172-8.

[26] D. Lu *et al.*, "Identifying potential risk genes for clear cell renal cell carcinoma with deep reinforcement learning," *Nature Communications,* vol. 16, no. 1, p. 3591, 2025/04/15 2025, doi: 10.1038/s41467-025-58439-5.

[27] X. Fu *et al.*, "BIDCell: Biologically-informed self-supervised learning for segmentation of subcellular spatial transcriptomics data," *Nature Communications,* vol. 15, no. 1, p. 509, 2024/01/13 2024, doi: 10.1038/s41467-023-44560-w.

[28] J. Tanevski, L. Vulliard, M. A. Ibarra-Arellano, D. Schapiro, F. J. Hartmann, and J. Saez-Rodriguez, "Learning tissue representation by identification of persistent local patterns in spatial omics data," *Nature Communications,* vol. 16, no. 1, p. 4071, 2025/04/30 2025, doi: 10.1038/s41467-025-59448-0.

[29] S. Yang *et al.*, "Organoids: The current status and biomedical applications," (in eng), *MedComm (2020),* vol. 4, no. 3, p. e274, Jun 2023, doi: 10.1002/mco2.274.

[30] C. W. Oomens, M. Maenhout, C. H. van Oijen, M. R. Drost, and F. P. Baaijens, "Finite element modelling of contracting skeletal muscle," (in eng), *Philos Trans R Soc Lond B Biol Sci,* vol. 358, no. 1437, pp. 1453-60, Sep 29 2003, doi: 10.1098/rstb.2003.1345.

[31] S. N. Makarov *et al.*, "Virtual Human Models for Electromagnetic Studies and Their Applications," (in eng), *IEEE Rev Biomed Eng,* vol. 10, pp. 95-121, 2017, doi: 10.1109/rbme.2017.2722420.

[32] B. Zhao *et al.*, "Heart-brain connections: Phenotypic and genetic insights from magnetic resonance images," *Science,* vol. 380, no. 6648, p. abn6598, 2023, doi: doi:10.1126/science.abn6598.

[33] J. Sacher and A. V. Witte, "Genetic heart–brain connections," *Science,* vol. 380, no. 6648, pp. 897-898, 2023, doi: doi:10.1126/science.adi2392.

[34] E. M. Klann *et al.*, "The Gut-Brain Axis and Its Relation to Parkinson's Disease: A Review," (in eng), *Front Aging Neurosci,* vol. 13, p. 782082, 2021, doi: 10.3389/fnagi.2021.782082.

[35] F. Isensee, P. F. Jaeger, S. A. A. Kohl, J. Petersen, and K. H. Maier-Hein, "nnU-Net: a self-configuring method for deep learning-based biomedical image segmentation," *Nat Methods,* vol. 18, no. 2, pp. 203-211, 2021/02/01 2021, doi: 10.1038/s41592-020-01008-z.

[36] F.-a. Wang *et al.*, "TMO-Net: an explainable pretrained multi-omics model for multi-task learning in oncology," *Genome Biology,* vol. 25, no. 1, p. 149, 2024/06/06 2024, doi: 10.1186/s13059-024-03293-9.

[37] J. Lee *et al.*, "BioBERT: a pre-trained biomedical language representation model for biomedical text mining," (in eng), *Bioinformatics,* vol. 36, no. 4, pp. 1234-1240, Feb 15 2020, doi: 10.1093/bioinformatics/btz682.
